# Characterization of wheat homeodomain-leucine zipper family genes and functional analysis of *TaHDZ5-6A* in drought tolerance in transgenic *Arabidopsis*

**DOI:** 10.1186/s12870-020-2252-6

**Published:** 2020-01-31

**Authors:** Shumin Li, Nan Chen, Fangfang Li, Fangming Mei, Zhongxue Wang, Xinxiu Cheng, Zhensheng Kang, Hude Mao

**Affiliations:** State Key Laboratory of Crop Stress Biology for Arid Areas, College of Plant Protection, Northwest A&F University, Yangling, 712100 Shaanxi China

**Keywords:** HD-zip gene family, Wheat, Phylogenetic relationships, Expression profiles, *TaHDZ5-6A*, Drought tolerance

## Abstract

**Background:**

Many studies in *Arabidopsis* and rice have demonstrated that HD-Zip transcription factors play important roles in plant development and responses to abiotic stresses. Although common wheat (*Triticum aestivum* L.) is one of the most widely cultivated and consumed food crops in the world, the function of the HD-Zip proteins in wheat is still largely unknown.

**Results:**

To explore the potential biological functions of *HD-Zip* genes in wheat, we performed a bioinformatics and gene expression analysis of the HD-Zip family. We identified 113 HD-Zip members from wheat and classified them into four subfamilies (I-IV) based on phylogenic analysis against proteins from *Arabidopsis*, rice, and maize. Most *HD-Zip* genes are represented by two to three homeoalleles in wheat, which are named as *TaHDZX_ZA*, *TaHDZX_ZB*, or *TaHDZX_ZD*, where X denotes the gene number and Z the wheat chromosome on which it is located. *TaHDZs* in the same subfamily have similar protein motifs and intron/exon structures. The expression profiles of *TaHDZ* genes were analysed in different tissues, at different stages of vegetative growth, during seed development, and under drought stress. We found that most *TaHDZ* genes, especially those in subfamilies I and II, were induced by drought stress, suggesting the potential importance of subfamily I and II *TaHDZ* members in the responses to abiotic stress. Compared with wild-type (WT) plants, transgenic *Arabidopsis* plants overexpressing *TaHDZ5-6A* displayed enhanced drought tolerance, lower water loss rates, higher survival rates, and higher proline content under drought conditions. Additionally, the transcriptome analysis identified a number of differentially expressed genes between *35S::TaHDZ5-6A* transgenic and wild-type plants, many of which are involved in stress response.

**Conclusions:**

Our results will facilitate further functional analysis of wheat *HD-Zip* genes, and also indicate that *TaHDZ5-6A* may participate in regulating the plant response to drought stress. Our experiments show that *TaHDZ5-6A* holds great potential for genetic improvement of abiotic stress tolerance in crops.

## Background

Changes to the transcriptome are achieved through the action of transcription factors (TFs), which repress or activate suites of genes to modulate plant growth and respond to environmental stimuli [1]. The HD-Zip family consists of a large number of transcription factors that seem be unique to the plant kingdom. HD-Zip proteins contain a Homeobox domain (HD) and an adjacent Leucine Zipper (LZ) motif [2]. The HD domain is responsible for specific DNA binding, whereas the LZ motif acts as a mediator to protein dimerization [2]. Based on the additional conserved motifs and their phylogenetic relationships, HD-Zip genes can be classified into four subfamilies (HD-Zip I, II, III, and IV) [2–5]. All subfamilies cotnain the LZ domain and are characterized by differences in the regions downstream of this domain. HD-Zip II subfamily proteins contain a conserved “CPSCE” motif located in the C-terminus, which is not found in HD-Zip I subfamily proteins [2]. HD-Zip III and IV subfamily proteins uniquely contain the extra conserved START and HD-SAD domains [2]. The HD-Zip III subfamily proteins are distinguished from those of HD-Zip IV by the presence and absence, respectively, of a C-terminal MEKHLA domain [2, 6].

In recent years, many efforts have been made to elucidate the functions of HD-Zip genes. Members of the HD-Zip family have been found to play pivotal roles in plant development and the adaption to environmental stresses. HD-Zip I subfamily proteins are mainly involved in the regulation of organ growth and development, de-etiolation, blue light signaling, and also in regulating the response to abiotic stresses [7–11]. For example, *ATHB7* and *ATHB12* are both sensitive to abscisic acid (ABA) and water deficit, and negatively regulate the ABA response in *Arabidopsis* [6]. *ATHB1* acts as a positive regulator to promote hypocotyl elongation [8] and to mediate the determination of leaf cell fate [9]. The *TaHDZipI-2* gene was shown to regulate flowering and spike development and improve frost tolerance in transgenic barley lines [10]. Additionally, wheat *TaHDZipI-3*, *− 4* and *− 5* genes are differentially expressed in response to abscisic acid (ABA), cold and drought treatment through binding to specific *cis*-elements [11]. HD-Zip II subfamily proteins participate in embryonic apical development, auxin signaling, and are also involved in light and abiotic stress responses [12–15]. In *Arabidopsis*, both *ATHB2/HAT4* and *HAT2* participate in auxin-mediated morphogenesis, and *ATHB2/HAT4* also regulates the leaf cell expansion and shade avoidance under red/far-red light [13, 14]. *OsHOX11* and *OsHOX27* are two rice HD-Zip II genes, and their expression is dramatically decreased upon exposure to drought in a drought-resistant cultivar [12]. Additionally, a sunflower HD-ZIP II gene, *HAHB10*, participates in the response to biotic stress [15].

HD-Zip III subfamily proteins have been reported to control embryogenesis, apical meristem development, vascular bundle development, morpho-physiological changes in roots and auxin transport, and leaf polarity [16–20]. *ATHB8* and *ATHB15* are thought to direct vascular development [17, 18]. CLV3 has been shown to interact with HD-Zip III members to regulate floral meristem activities [19], and KANADI interacts with HD-Zip III genes to control lateral root development [20]. *PopREVOLUTA (PRE)*, a class III HD-Zip gene in poplar, is involved in the growth of cambia and secondary vascular tissues [16]. HD-Zip IV subfamily proteins are integral to growth and development of trichome, cuticle, and root tissues, as well as epidermal cell differentiation [21–24]. In *Arabidopsis*, *GL2* regulates trichome expansion and root hair differentiation [22], and *PDF2* plays a vital role in epidermal cells to control normal development of the floral organs [21]. *OCL4* (*OUTER CELL LAYER4*) encodes a maize HD-Zip IV transcription factor that inhibits trichome development and influences anther cell division in maize [23]. In addition, recent studies have demonstrated that overexpression of *AtHDG11*, an HD-Zip IV gene, increases drought tolerance in *Arabidopsis*, tobacco, rice, sweet potato, and cotton [24].

Bread wheat (*Triticum aestivum*; 2*n* = 6x = 42; AABBDD) is an integral global food crop [25, 26]. The modern bread wheat genome is the result of two allopolyploidization events with three genomes. First, the A genome donor (*T. urartu*, AA; 2*n* = 14) hybridized with the B genome donor (*A. speltoides*, SS; 2*n* = 14). This event, which occurred ~ 0.2 Mya, produced the allotetraploid *T. turgidum* L. (AABB). Second, this AABB donor hybridized with the D genome donor (*A. tauschii* ~ 9000 ya. This resulted in the allohexaploid wheat *T. aestivum* (AABBDD) [27, 28], which has a large (> 17 Gb) and composite genome, making genomic studies difficult. Because of wheat’s importance globally, extensive research has been conducted to sequence and annotate its genome [25, 26, 29–33]. Recent efforts have sequenced isolated chromosome arms and constructed a draft sequence of the hexaploid wheat genome (IWGSC, 2018). However, compared with *Arabidopsis* and rice, there are fewer studies of the HD-Zip family in wheat. To date, only five genes encoding HD-Zip subfamily I members (*TaHDZipI-1* to *TaHDZipI-5*) have been isolated and partially characterized from wheat [10, 11, 34]. Although some *HD-Zip* genes have been functional studied in wheat, the reports of their genome organization, structure and phylogenetic relationships are limitted, especially for *HD-Zip* genes involved in drought stress response.

In a previous study, 46 wheat *HD-Zip* genes were identified [35], which is not consistent with the large genome of wheat. Thus, a further survey of the *HD-Zip* gene family should be conducted using the most current version of the wheat genome. Here, we present a genome-wide identification and analysis of the *HD-Zip* genes from wheat and show the phylogenetic relationships among the wheat genes and to those from *Arabidopsis* and other plants. We performed gene expression analyses to characterize the expression profiles of *HD-Zip* genes in various organs/tissues and in response to drought stress. We then performed functional analysis of a drought- induced *HD-Zip I* gene, *TaHDZ5-6A*, by investigating drought stress tolerance and physiological traits in transgenic *Arabidopsis* plants. Finally, we propose a putative mechanism by which *TaHDZ5-6A* enhances drought tolerance in transgenic *Arabidopsis* plants. Our results provide a basis for the further functional analysis of the wheat *HD-Zip* gene family.

## Results

### Identification of the HD-zip gene family in wheat

Wheat genome data used in this study were downloaded from the Chinese Spring IWGSC RefSeq v1.1 reference genome assembly (https://wheat-urgi.versailles.inra. fr/). We firstly converted the wheat genome into a local BLAST database using the UNIX pipeline. Then, we used 90 *Arabidopsis* and rice HD-Zip protein sequences to perform a BLAST search (BLASTP) against this local blast database using cut-off *E*-value <1e-10. After remove the all redundant sequences using CD-hit program, the rest of protein sequences were further subjected to identify the HD domain and LZ motif using the Simple Modular Architecture Research Tool (SMART; http://smart.embl-heidelberg.de/smart/set_mode.cgi? NORMAL = 1). In a recently study, a total of 46 *HD-Zip* genes were identified in wheat by a genome-wide bioinformatic survey [35]. In this study, we further identified 67 additional *HD-Zip* genes in wheat latest genome and extended the total member to 113. Based on the genomic position information, 113 *HD-Zip* genes were located across all the 21 wheat chromosomes, ranging from 3 to 8 per chromosome. Chromosome 5A/B/D have the most HD-Zip genes (24 total, 8 per chromosome), followed by chromosome 4A/B/D (18 total, 6 per chromosome) (Table 1; Additional file 1: Figure S1). Acording to their phylogenetic relationship, the 113 HD-Zip proteins were grouped into 40 homoeologous clusters, and the members in each of 39 clusters were assigned to A, B or D sub-genomes. Finally, We designated wheat *HD-Zip* genes as *TaHDZX_ZA*, *TaHDZX_ZB*, or *TaHDZX_ZD*, where X denotes the gene number and Z the wheat chromosome where it is located. The detailed information of HD-Zip family genes in wheat, including nomenclature proposed in the previous study [35] was listed in Table 1. As shown in Table 1, the identified *HD-Zip* genes in wheat encode proteins ranging from 192 (TaHDZ12-6D) to 890 (TaHDZ35-1B) amino acids (aa) in length with an average of 501 aa. Furthermore, the computed molecular weights of these HD-Zip proteins ranged from 20.88 (TaHDZ12-6D) to 96.02 (TaHDZ35-1B) kDa. The theoretical pI of the deduced HD-Zip proteins ranged from 4.59 (TaHDZ5-6A) to 9.79 (TaHDZ12-6D).

**Table 1:** Detail information of wheat *HD-Zip* genes

Name	Protein id	chr	Start	End	Number of amino acids	Molecular weight	Theoretical pI	group	Previous nomenclatur e[35]
TaHDZ1-4A	TraesCS4A02G405800	chr4A	678,872,316	678,873,272	285	31,206.22	4.79	I	TaHDZ16-A/Traes_4AL_99A941299
TaHDZ1-4B	TraesCS4B02G305300	chr4B	593,422,292	593,423,732	316	35,099.79	4.99	I	TaHDZ16-B/Traes_4BL_ECD20BE67
TaHDZ1-4D	TraesCS4D02G303500	chr4D	471,961,107	471,962,520	316	34,919.58	4.98	I	
TaHDZ2-5A	TraesCS5A02G249000	chr5A	463,452,047	463,453,063	270	28,917.9	4.77	I	
TaHDZ2-5B	TraesCS5B02G246700	chr5B	428,461,602	428,462,604	269	28,884.88	4.7	I	TaHDZ22-B/Traes_5BL_5DE02D63E
TaHDZ2-5D	TraesCS5D02G256200	chr5D	362,547,609	362,548,803	270	28,909.87	4.7	I	
TaHDZ3-4A	TraesCS4A02G016600	chr4A	11,296,831	11,299,294	338	35,973.87	6.08	I	TaHDZ15-A/Traes_4AS_F04DD4409
TaHDZ3-4B	TraesCS4B02G287600	chr4B	571,047,644	571,049,696	331	36,494.59	6.17	I	TaHDZ15-B/Traes_4BL_BE3E058A6
TaHDZ3-4D	TraesCS4D02G286400	chr4D	457,074,633	457,076,703	330	36,411.5	6.17	I	TaHDZ15-D/Traes_4DL_88ABAD6C0
TaHDZ4-5A	TraesCS5A02G199300	chr5A	404,177,302	404,178,633	300	32,713.28	4.86	I	
TaHDZ4-5B	TraesCS5B02G197700	chr5B	356,597,007	356,598,447	299	32,566.01	4.86	I	TaHDZ21-B/Traes_5BL_028D02DF6
TaHDZ4-5D	TraesCS5D02G205000	chr5D	310,677,920	310,679,395	299	32,637.21	4.93	I	
TaHDZ5-6A	TraesCS6B02G321100	chr6B	568,257,664	568,259,128	343	37,502.12	4.59	I	TaHDZ24-A/Traes_6AL_36AB0312C
TaHDZ5-6D	TraesCS6D02G272000	chr6D	380,799,345	380,800,814	374	41,012.34	4.82	I	TaHDZ24-D/Traes_6DL_FF4C8C4AB
TaHDZ6-5A	TraesCS5A02G316800	chr5A	527,904,954	527,905,823	249	27,500.54	4.97	I	
TaHDZ6-5B	TraesCS5B02G317400	chr5B	501,464,138	501,465,028	249	27,502.62	5.02	I	TaHDZ20-B/Traes_5BL_9C32B27E2
TaHDZ6-5D	TraesCS5D02G323100	chr5D	415,272,394	415,273,274	247	27,246.32	5.09	I	TaHDZ20-D/Traes_5DL_96F9EED93
TaHDZ7-2A	TraesCS2A02G389400	chr2A	637,984,504	637,985,699	265	29,697.25	5.12	I	TaHDZ8-A/Traes_2AL_BFB0C6D4C
TaHDZ7-2B	TraesCS2B02G407600	chr2B	578,605,119	578,605,996	260	29,074.51	5.2	I	TaHDZ8-B/Traes_2BL_B69300543
TaHDZ7-2D	TraesCS2D02G387300	chr2D	492,676,386	492,677,218	243	26,899.14	5.04	I	
TaHDZ8-6A	TraesCS6A02G240400	chr6A	451,659,958	451,660,731	221	24,977.72	5.27	I	
TaHDZ8-6B	TraesCS6B02G284300	chr6B	512,447,547	512,448,327	226	25,584.39	5.09	I	
TaHDZ8-6D	TraesCS6D02G222600	chr6D	314,056,149	314,056,946	225	25,536.5	5.65	I	TaHDZ26-D/Traes_6DS_D281B7D32
TaHDZ9-4A	TraesCS4A02G040600	chr4A	34,026,753	34,027,976	231	25,530.54	6.24	I	TaHDZ17-A/Traes_4AS_1EA23DE08
TaHDZ9-4B	TraesCS4B02G261600	chr4B	530,519,687	530,520,929	233	25,576.61	6.04	I	TaHDZ17-B/Traes_4BL_BE10705D5
TaHDZ9-4D	TraesCS4D02G261600	chr4D	432,748,587	432,749,803	234	25,708.82	6.24	I	TaHDZ17-D/Traes_4DL_4798D0BBD
TaHDZ10-2B	TraesCS2B02G405700	chr2B	573,974,813	573,975,706	221	24,267	7.7	I	TaHDZ7-B/Traes_2BL_419CEED79
TaHDZ10-2D	TraesCS2D02G385500	chr2D	490,117,422	490,118,593	247	27,340.52	7.17	I	
TaHDZ11-2A	TraesCS2A02G188500	chr2A	153,452,292	153,453,239	238	25,785.75	7.12	I	
TaHDZ11-2B	TraesCS2B02G218800	chr2B	208,210,912	208,211,847	238	25,858.89	7.12	I	TaHDZ6-B/Traes_2BS_BD0ED621D
TaHDZ11-2D	TraesCS2D02G199200	chr2D	148,855,464	148,856,413	238	25,755.72	6.76	I	TaHDZ6-D/Traes_2DS_20F748657
TaHDZ12-6A	TraesCS6A02G120300	chr6A	91,924,600	91,925,404	203	22,045.89	9.64	II	
TaHDZ12-6B	TraesCS6B02G148700	chr6B	149,551,263	149,551,965	209	22,641.46	9.41	II	
TaHDZ12-6D	TraesCS6D02G110400	chr6D	76,042,794	76,043,516	192	20,888.56	9.79	II	TaHDZ27-D/Traes_6DS_F00EB2E01
TaHDZ13-6A	TraesCS6A02G120600	chr6A	92,054,391	92,055,152	226	24,538.57	8.72	II	TaHDZ25-A/Traes_6AS_3E534A2C1
TaHDZ13-6B	TraesCS6B02G149000	chr6B	149,773,307	149,774,051	220	24,273.43	9.32	II	
TaHDZ13-6D	TraesCS6D02G110600	chr6D	76,064,358	76,065,121	225	24,759.74	8.38	II	TaHDZ25-D/Traes_6DS_17B737547
TaHDZ14-7A	TraesCS7A02G423800	chr7A	616,552,387	616,553,258	219	24,421.47	9.25	II	TaHDZ28-A/Traes_7AL_44206BE21
TaHDZ14-7B	TraesCS7B02G326300	chr7B	579,825,401	579,826,290	223	24,708.84	9.47	II	
TaHDZ14-7D	TraesCS7D02G417700	chr7D	536,514,689	536,515,582	222	24,526.65	9.61	II	TaHDZ28-D/Traes_7DL_2FE5181AF
TaHDZ15-1A	TraesCS1A02G372900	chr1A	549,301,452	549,303,005	305	33,195.14	6.27	II	
TaHDZ15-1B	TraesCS1B02G393100	chr1B	626,097,677	626,098,967	306	33,662.73	6.68	II	TaHDZ3-B/Traes_1BL_BCA60D8B6
TaHDZ15-1D	TraesCS1D02G379700	chr1D	455,759,848	455,761,548	304	33,117.06	6.67	II	
TaHDZ16-4A	TraesCS4A02G059600	chr4A	56,143,977	56,145,391	275	29,373.91	6.67	II	
TaHDZ16-4B	TraesCS4B02G235300	chr4B	491,464,360	491,465,839	277	29,618.16	6.67	II	TaHDZ18-B/Traes_4BL_78DD63002
TaHDZ16-4D	TraesCS4D02G236600	chr4D	398,669,293	398,670,678	274	29,332.85	6.67	II	
TaHDZ17-3B	TraesCS3B02G000100	chr3B	213,438	214,466	228	25,282.62	8.64	II	TaHDZ10-B/TRAES3BF043500070CFD
TaHDZ17-3D	TraesCS3D02G009700	chr3D	3,294,033	3,295,097	226	24,874.23	9.24	II	TaHDZ10-D/Traes_3DS_7CCB5ECD2
TaHDZ18-5A	TraesCS5A02G232700	chr5A	448,090,083	448,091,497	351	36,587.01	7.01	II	
TaHDZ18-5B	TraesCS5B02G231300	chr5B	407,816,831	407,818,230	355	37,049.33	6.27	II	TaHDZ19-B/Traes_5BL_4A3874701
TaHDZ18-5D	TraesCS5D02G235300	chr5D	344,398,948	344,400,554	339	35,808.01	6.49	II	
TaHDZ19-3A	TraesCS3A02G231600	chr3A	432,374,494	432,377,827	222	24,782.22	9.17	II	
TaHDZ19-3B	TraesCS3B02G260800	chr3B	418,718,384	418,721,923	222	24,704.13	9.42	II	TaHDZ11-B/TRAES3BF026400090CFD
TaHDZ19-3D	TraesCS3D02G221800	chr3D	302,265,801	302,269,312	222	24,776.24	9.27	II	
TaHDZ20-1A	TraesCS1A02G219200	chr1A	387,840,646	387,841,766	329	35,072.38	8.71	II	TaHDZ4-A/Traes_1AL_1444D461A
TaHDZ20-1B	TraesCS1B02G232700	chr1B	418,105,692	418,106,801	327	34,775.13	8.88	II	
TaHDZ20-1D	TraesCS1D02G220900	chr1D	308,459,300	308,460,410	326	34,691.06	8.89	II	
TaHDZ21-2A	TraesCS2A02G415900	chr2A	672,415,211	672,416,239	227	25,675.08	8.84	II	TaHDZ9-A/Traes_2AL_EF9549D16
TaHDZ21-2B	TraesCS2B02G434900	chr2B	624,891,321	624,892,387	230	26,196.48	8.84	II	TaHDZ9-B/Traes_2BL_02479C76A
TaHDZ21-2D	TraesCS2D02G412900	chr2D	527,546,793	527,547,829	230	25,914.27	8.84	II	TaHDZ9-D/Traes_2DL_67F1183B2
TaHDZ22-4A	TraesCS4A02G382400	chr4A	660,653,849	660,655,014	344	36,296.63	9.53	II	
TaHDZ23-7A	TraesCS7A02G083800	chr7A	48,525,735	48,526,646	266	28,231.91	9.16	II	TaHDZ14-A/Traes_4AL_822582A19
TaHDZ23-7D	TraesCS7D02G079000	chr7D	46,711,457	46,712,351	269	28,558.14	9.26	II	
TaHDZ24-3A	TraesCS3A02G312800	chr3A	552,611,093	552,615,178	874	94,707.86	6.06	III	
TaHDZ24-3B	TraesCS3B02G159100	chr3B	154,220,609	154,224,807	841	91,388.76	5.95	III	
TaHDZ24-3D	TraesCS3D02G141500	chr3D	103,645,450	103,649,526	845	91,803.17	5.92	III	
TaHDZ25-1A	TraesCS1A02G157500	chr1A	279,733,261	279,742,276	840	92,041.09	5.65	III	TaHDZ1-A/Traes_1AL_0BE456AC0
TaHDZ25-1B	TraesCS1B02G173900	chr1B	311,419,246	311,427,777	840	92,056.14	5.65	III	TaHDZ1-B/Traes_1BL_43408C9B0
TaHDZ25-1D	TraesCS1D02G155200	chr1D	217,636,182	217,644,644	606	66,231.68	6.31	III	
TaHDZ26-4B	TraesCS4B02G385200	chr4B	664,152,394	664,159,763	839	91,509.33	5.55	III	
TaHDZ26-4D	TraesCS4D02G359600	chr4D	506,968,051	506,975,377	838	91,591.45	5.61	III	
TaHDZ27-5A	TraesCS5A02G375800	chr5A	573,645,493	573,651,017	862	93,733.6	6.09	III	
TaHDZ27-5B	TraesCS5B02G378000	chr5B	556,177,511	556,182,640	862	93,776.77	6.09	III	
TaHDZ27-5D	TraesCS5D02G385300	chr5D	454,414,315	454,419,575	862	93,676.55	6.09	III	
TaHDZ28-5A	TraesCS5A02G043400	chr5A	39,845,363	39,851,741	846	91,939.04	6.1	III	
TaHDZ28-5B	TraesCS5B02G047200	chr5B	53,246,630	53,252,235	879	95,339.96	6.61	III	TaHDZ23-B/Traes_5BS_360DD5644
TaHDZ28-5D	TraesCS5D02G052300	chr5D	50,483,246	50,488,859	883	95,733.49	6.73	III	TaHDZ23-D/Traes_5DS_50846FD0C
TaHDZ29-3A	TraesCS3A02G325800	chr3A	571,174,922	571,179,414	683	74,860.15	7.92	IV	
TaHDZ29-3B	TraesCS3B02G354900	chr3B	565,265,645	565,270,023	683	74,621.82	8.17	IV	TaHDZ13-B/TRAES3BF075200070CFD
TaHDZ29-3D	TraesCS3D02G319200	chr3D	433,133,441	433,137,721	683	74,697.92	7.58	IV	TaHDZ13-D/Traes_3DL_8AAFB7B06
TaHDZ30-4A	TraesCS4A02G231300	chr4A	540,600,328	540,604,473	770	83,653.94	6.99	IV	
TaHDZ30-4B	TraesCS4B02G084700	chr4B	83,052,927	83,058,776	788	85,693.54	6.36	IV	
TaHDZ30-4D	TraesCS4D02G082600	chr4D	56,025,816	56,030,159	805	87,663.69	6.79	IV	
TaHDZ31-5A	TraesCS5A02G330200	chr5A	539,504,162	539,509,761	744	80,684.05	6.1	IV	
TaHDZ31-5B	TraesCS5B02G330300	chr5B	514,678,511	514,683,625	751	81,666.17	6.02	IV	
TaHDZ31-5D	TraesCS5D02G335900	chr5D	425,465,587	425,471,148	753	82,053.73	5.8	IV	
TaHDZ32-3A	TraesCS3A02G305300	chr3A	541,243,574	541,247,038	761	82,154.35	7.49	IV	
TaHDZ32-3B	TraesCS3B02G331200	chr3B	536,388,767	536,392,301	755	81,462.58	7.8	IV	TaHDZ12-B/TRAES3BF023000040CFD
TaHDZ32-3D	TraesCS3D02G296500	chr3D	410,412,719	410,416,215	778	84,493.39	9.14	IV	
TaHDZ33-6A	TraesCS6A02G324500	chr6A	558,785,948	558,788,800	685	75,094.44	6.33	IV	
TaHDZ33-6B	TraesCS6B02G354900	chr6B	623,081,402	623,084,291	697	75,242.66	6.48	IV	
TaHDZ33-6D	TraesCS6D02G304300	chr6D	413,269,725	413,272,565	685	75,009.28	6.25	IV	
TaHDZ34-7A	TraesCS7A02G167900	chr7A	124,067,724	124,070,795	725	79,209	6.13	IV	
TaHDZ34-7B	TraesCS7B02G072700	chr7B	80,658,897	80,662,738	730	79,513.39	6.19	IV	
TaHDZ34-7D	TraesCS7D02G168700	chr7D	119,565,615	119,569,016	732	79,795.75	6.22	IV	
TaHDZ35-1A	TraesCS1A02G193400	chr1A	350,395,750	350,401,039	873	94,078.96	5.68	IV	
TaHDZ35-1B	TraesCS1B02G208400	chr1B	377,965,083	377,969,806	890	96,021.18	5.57	IV	
TaHDZ35-1D	TraesCS1D02G197300	chr1D	278,105,002	278,110,971	883	95,091.14	5.64	IV	TaHDZ2-D/Traes_1DL_9FB53E48A
TaHDZ36-6A	TraesCS6A02G255800	chr6A	474,327,845	474,334,088	804	86,379.32	5.51	IV	
TaHDZ36-6B	TraesCS6B02G269700	chr6B	485,537,128	485,542,736	804	86,399.39	5.51	IV	
TaHDZ36-6D	TraesCS6D02G237000	chr6D	335,062,906	335,068,647	804	86,465.43	5.46	IV	
TaHDZ37-2A	TraesCS2A02G401200	chr2A	654,907,253	654,913,197	798	85,781.8	5.54	IV	
TaHDZ37-2B	TraesCS2B02G419200	chr2B	600,740,018	600,745,678	785	84,370.1	5.92	IV	
TaHDZ37-2D	TraesCS2D02G398600	chr2D	511,688,028	511,693,305	784	84,013.06	5.87	IV	TaHDZ5-D/Traes_2DL_036F2A3FC
TaHDZ38-5A	TraesCS5A02G314400	chr5A	524,906,817	524,907,230	849	90,375.05	5.7	IV	
TaHDZ38-5B	TraesCS5B02G315100	chr5B	497,213,945	497,219,576	849	90,378.01	5.7	IV	
TaHDZ38-5D	TraesCS5D02G320600	chr5D	412,738,548	412,744,058	849	90,401.09	5.7	IV	
TaHDZ39-7A	TraesCS7A02G308400	chr7A	436,693,693	436,698,045	796	85,517.17	5.99	IV	
TaHDZ39-7B	TraesCS7B02G208600	chr7B	381,824,085	381,829,068	798	85,647.27	5.99	IV	
TaHDZ39-7D	TraesCS7D02G305200	chr7D	386,725,334	386,730,432	796	85,592.19	5.99	IV	
TaHDZ40-2A	TraesCS2A02G474000	chr2A	715,337,511	715,343,047	777	83,524.33	5.59	IV	
TaHDZ40-2B	TraesCS2B02G497500	chr2B	694,054,407	694,060,937	775	83,454.3	5.65	IV	
TaHDZ40-2D	TraesCS2D02G473700	chr2D	577,152,396	577,158,953	776	83,438.3	5.59	IV	

### Phylogenetic analysis of HD-zip gene family

**Fig. 1:**
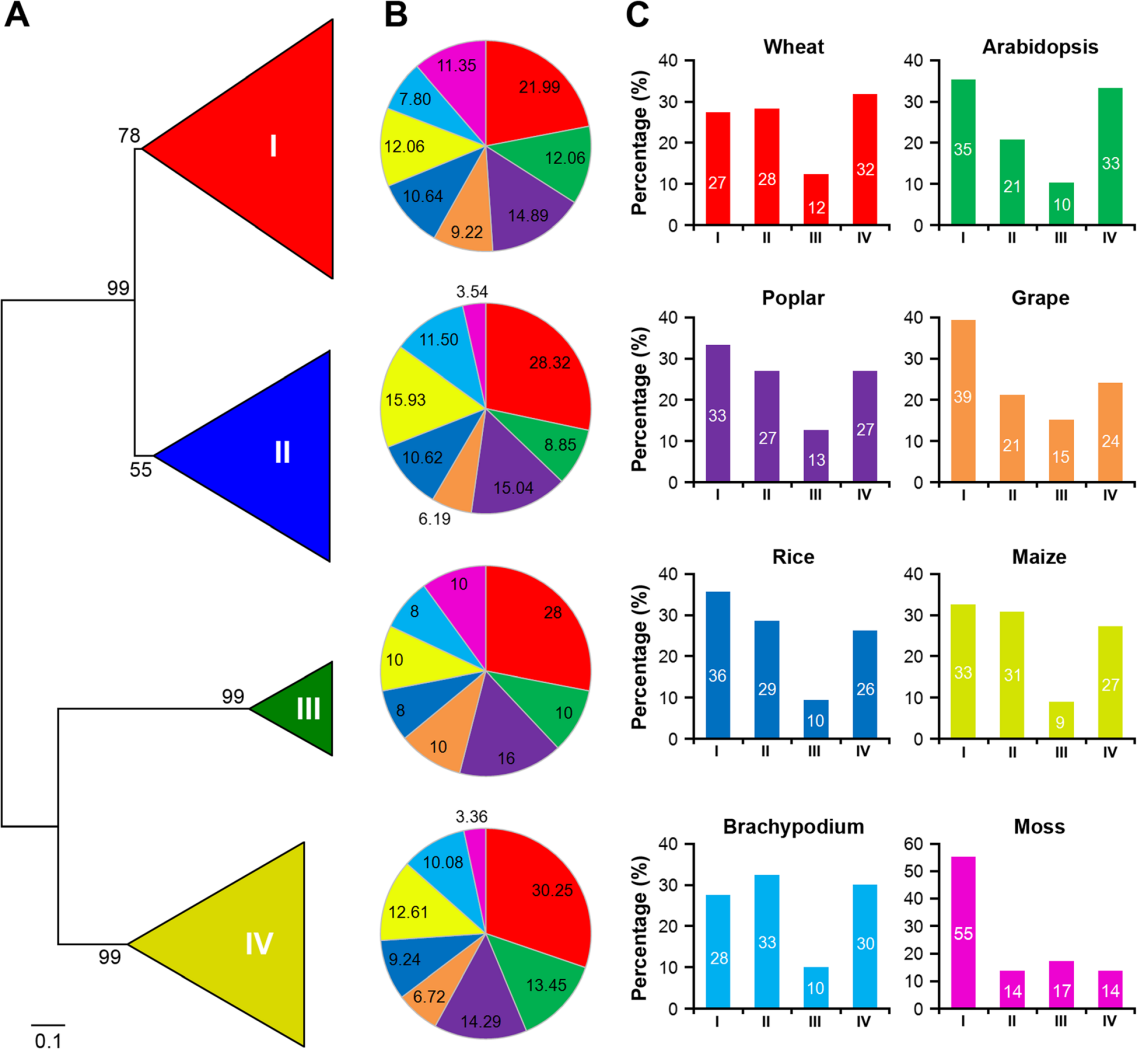
Phylogeny and distribution of HD-Zip proteins from eight plant species. a Phylogenetic tree of HD-Zip proteins from *Arabidopsis*, *Populus*, *Vitis*, wheat, rice, maize, *Brachypodium*, and moss. Phylogeny was constructed by PhyML using maximum likelihood analysis. Bootstrap support values as percentage, are shown on selected major branches. The bar indicates substitutions per site; b Percentage representation of HD-Zips across the eight plant species within each subfamily; c Percentage representation of distributions for HD-Zips within each plant species

Our study aimed to understand the phylogenetic relationships between plant HD-Zip proteins. We began by identification of *HD-Zip* genes from seven other plant species with varying levels of complexity for which entire genomes were accessible, including *Chlamydomonas reinhardtii*, *Physcomitrella patens*, the monocotyledonous angiosperms *Brachypodium distachyon*, *Oryza sativa*, and *Zea mays*, and the dicotyledonous angiosperms *Arabidopsis thaliana*, *Populus trichocarpa*, and *Vitis vinifera*. From this analysis, we found that the *HD-Zip* gene family seems to be restricted to land plants; all genomes except that of the algae contained genes for HD-Zip proteins. We then analyzed their evolutionary relationships using full-length HD-Zip proteins from eight land plant species to construct a neighbour-joining phylogenetic tree. Acordingly, the phylogenetic tree was divided into four well-conserved subfamilies, designated as HD-Zip I to IV (Fig. 1a). The phylogenetic tree also revealed the species-biased distribution of these plant HD-Zip proteins (Fig. 1b). HD-Zip I members consisted of the largest subfamily in the plant species except for *Brachypodium distachyon* and wheat, where HD-Zip II and IV were the largest respectively. In contrast, HD-Zip III subfamily composed of the fewest HD-Zip members except for moss (Fig. 1c). Subfamily I included 31 *TaHDZ* genes, grouped into 11 clusters (*TaHDZ1-4A/B/D*, *TaHDZ2-5A/B/D*, *TaHDZ3-4A/B/D*, *TaHDZ4-5A/B/D*, *TaHDZ5-6A/D*, *TaHDZ6-5A/B/D*, *TaHDZ7-2A/B/D*, *TaHDZ8-6A/B/D*, *TaHDZ9-4A/B/D*, *TaHDZ10-2B/D*, and *TaHDZ11-2A/B/D*); Similarly, subfamily II embraces 31 *TaHDZs*, grouped into 12 clusters (*TaHDZ12-6A/B/D*, *TaHDZ13-6A/B/D*, *TaHDZ14-7A/B/D*, *TaHDZ15-1A/B/D*, *TaHDZ16-4B/D*, *TaHDZ17-3B/D*, *TaHDZ18-5A/B/D*, *TaHDZ19-3A/B/D*, *TaHDZ20-1A/B/D*, *TaHDZ21-2A/B/D*, *TaHDZ22-4A*, and *TaHDZ23-7A/D*); While subfamily III is the smallest, and contained 14 *TaHDZs*, which grouped into 5 clusters (*TaHDZ24-3A/B/D*, *TaHDZ25-1A/B/D*, *TaHDZ26-4B/D*, *TaHDZ27-5A/B/D*, and *TaHDZ28-5A/B/D*); subfamily IV contained 36 *TaHDZs*, and grouped into 12 clusters (*TaHDZ29-3A/B/D*, *TaHDZ30-4A/B/D*, *TaHDZ31-5A/B/D*, *TaHDZ32-3A/B/D*, *TaHDZ33-6A/B/D*, *TaHDZ34-7A/B/D*, *TaHDZ35-1A/B/D*, *TaHDZ36-6A/B/D*, *TaHDZ37-2A/B/D*, *TaHDZ38-5A/B/D*, *TaHDZ39-7A/B/D*, and *TaHDZ40-2A/B/D*) (Table 1).

**Fig. 2:**
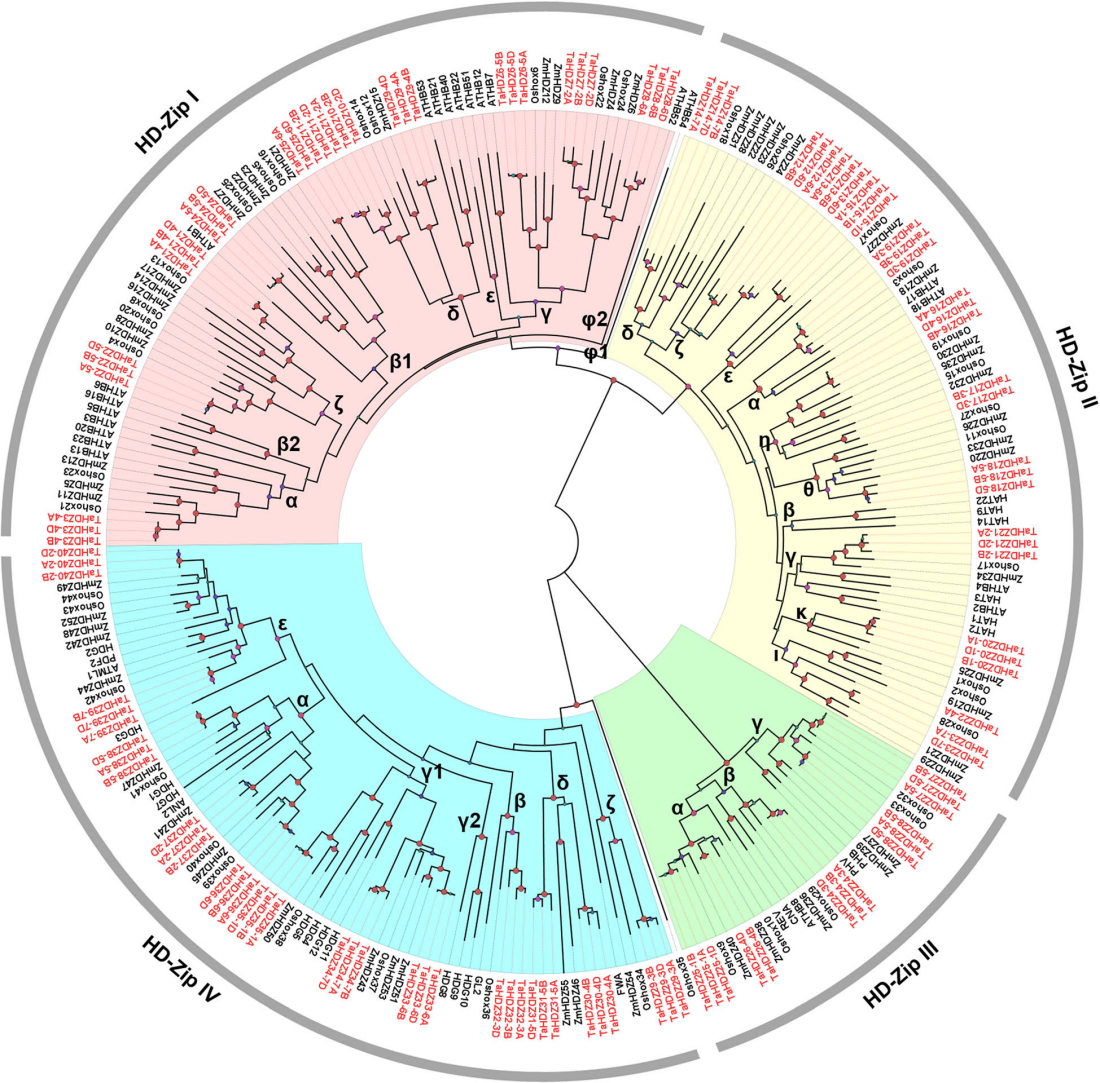
The phylogenetic tree of HD-Zip proteins from wheat, *Arabidopsis*, maize and rice. Members of the *HD-zip* genes from wheat are marked in red. Two-letter prefixes for sequence identifiers indicate species of origin. Ta, *Triticum aestivum*; At, *Arabidopsis thaliana*; Os, *Oryza sativa*; Zm, *Zea mays*. The tree was constructed using the Neighbor-Joining algorithm with 1000 bootstrap based on the full length sequences of HD-Zip proteins. The HD-Zip proteins are grouped into four distinct groups

To clarify the paralog and ortholog relationships of wheat HD-Zip members, we further divided each subfamily into subclasses. According to this reshaped phylogenic tree (Fig. 2), each subfamily contain the HD-Zip proteins from *Arabidopsis*, rice, and wheat, suggesting that these subfamilies were appeared before the dicot-monocot split. Consistent with the nomenclature in previous studies of *Arabidopsis* and rice [36], HD-Zip I subfamily was divided into seven subclasses, i.e., α, β, γ, δ, ε, φ and ζ (Fig. 2). Clade ε and φ contains only sequences from *Arabidopsis*. Clade ζ contains sequences from both rice and wheat, with no members in *Arabidopsis*, suggesting the gene loss in *Arabidopsis* during the long period of evolution of this group. The HD-Zip II subfamily was divided into ten subclasses, from α to κ, according to Hu et al. (2012) [37]. Clade β contains only sequences from *Arabidopsis*. Clade α and γ contains sequences from both rice, wheat, and *Arabidopsis*. While the other clades only contains sequences from rice and wheat. The HD-Zip III subfamily was only divided into three subclades, designated as clade α, β and γ, consistent with the previous studies [37]. Each clade contains sequences from both rice, wheat, and *Arabidopsis*. The HD-Zip IV subfamily was also divided into six subclades, designated clade α, β, γ, δ, ε and ζ as in a previous study [37]. Clade δ excluded genes from rice and *Arabidopsis*, while clade ζ included only sequences from rice and wheat. Eudicot- and monocot-specific clustering patterns of *HD-Zip genes* emerged when tree topology was examined. This pattern may reflect evolutionary history of these subgroups: *HD-Zip* genes in eudicots were likely retained after they diverged from monocots and then expanded.

### Gene structure and motif composition analysis

**Fig. 3:**
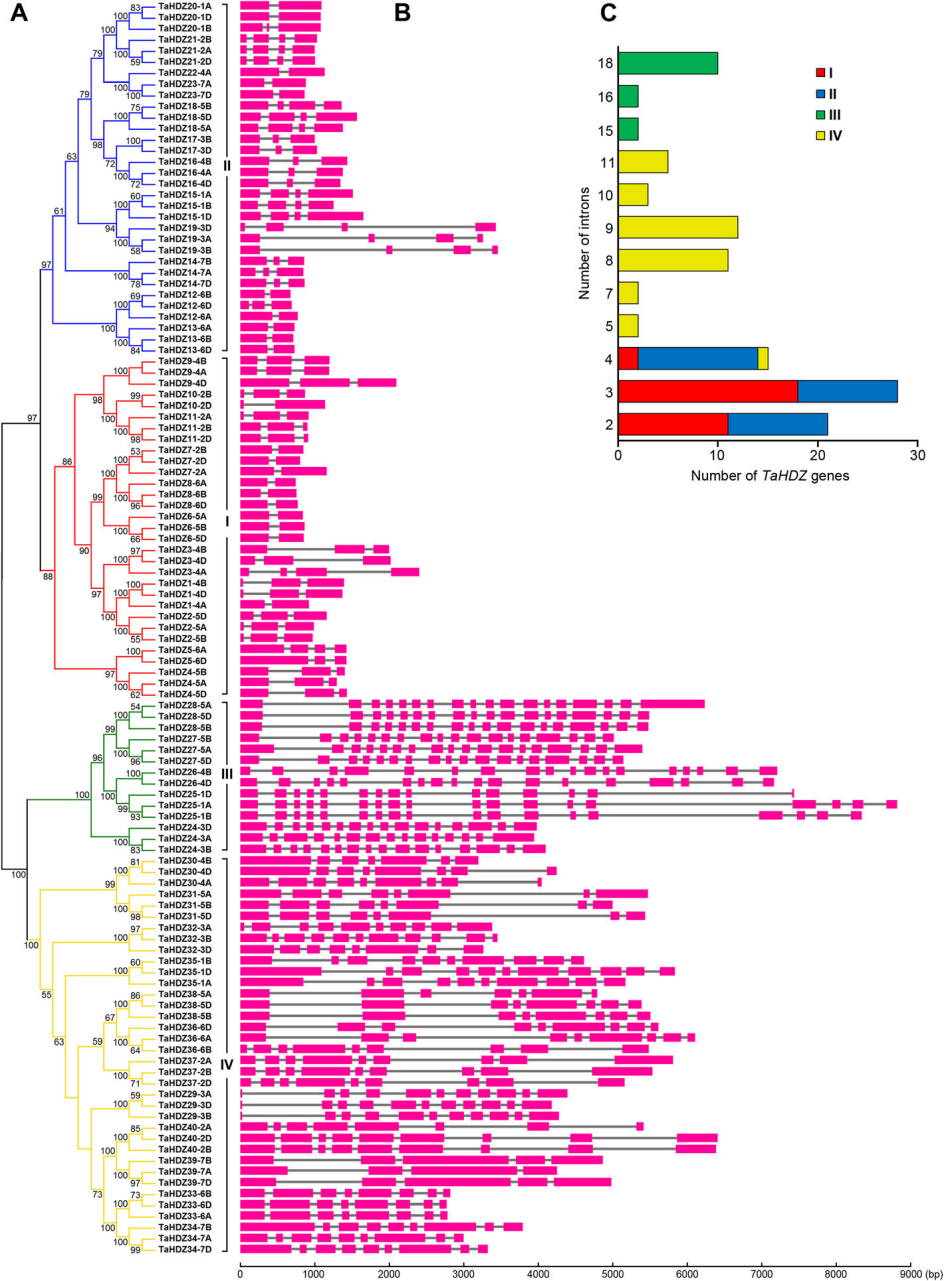
Phylogenetic relationships and gene structures of wheat *HD-Zip* genes. **a** Phylogenetic tree of 113 full length HD-Zip proteins from wheat were constructed by MEGA 6.0 using the Neighbour-Joining (NJ) method with 1000 bootstrap values. **b** Exon/intron structures of wheat *HD-Zip* genes. Exons and introns are represented by purple boxes and grey lines, respectively. **c** The distribution of intron numbers between four distinct HD-Zip subfamily of wheat

Exon-intron structural divergence can play an important role in the evolution of multiple gene families [38]. We constructed a phylogenetic tree using only the 113 full-length wheat HD-Zip protein sequences fo further examine patterns in wheat. We found that wheat HD-Zip proteins also fell into the four subfamilies described previously (Fig. 3a). We further mapped the exon/intron organization in the coding regions of each *TaHDZ* gene. Specifically, 21 *TaHDZ* genes had two introns, 28 had three introns, 15 had four introns, two had five introns, two had seven introns, 11 had eight introns, 12 had nine introns, three had 10 introns, five had 11 introns, two had 15 introns, two had 16 introns, and 10 had 18 introns (Fig. 3b, c). In general, orthologous genes are highly conserved with respect to gene structure, and this conservation is sufficient to reveal their evolutionary relationships [38]. In wheat, *HD-Zip* genes within the same subfamily shared similar gene structures (intron number and exon length), especially the members of the HD-Zip I and HD-Zip III subfamilies, i.e., HD-Zip I genes mainly had two or three introns in their gene regions, and HD-Zip III genes mainly had 18 introns. However, the exon/intron compositions in HD-Zip II and IV genes were more variable, i.e., HD-Zip II members possessed two to four introns, and the number of introns in HD-Zip IV family members varied from 4 to 11 (Fig. 3b, c).

The allohexaploid bread wheat genome is known to have formed by fusion of the *T. urartu* (subgenome A), *Aegilops speltoides* (subgenome B), and *A. tauschii* (subgenome D) genomes prior to several hundred thousand years ago. A majority (60.1–61.3%) of genes in the A, B, and D sub-genomes have orthologs in all the related diploid genomes. To deeply understand the intron gain or loss for homeologous *TaHDZ* genes in wheat, the intron/exon structures of *TaHDZ* genes that clustered together based on the phylogenetic tree were compared. Among these, fourteen clusters showed changes in their intron/exon structure, including *TaHDZ1-4A/B/D*, *TaHDZ3-4A/B/D*, *TaHDZ5-6A/D*, *TaHDZ10-2B/D*, *TaHDZ12-6A/B/D*, *TaHDZ20-1A/B/D*, *TaHDZ24-3A/B/D*, *TaHDZ25-1A/B/D*, *TaHDZ30-4A/B/D*, *TaHDZ32-3A/B/D*, *TaHDZ35-1A/B/D*, *TaHDZ38-5A/B/D*, *TaHDZ39-7A/B/D*, and *TaHDZ40-2A/B/D* (Fig. 3b). Because there are many orthologs in the wheat A, B, and D sub-genomes, intron gain/loss of these orthologs significantly increases the transcriptome and proteome complexity in wheat.

To further examine the diverse structurse of wheat HD-Zip proteins, the conserved motifs were identified by searching the SALAD database along with subsequent annotation with InterPro (Additional file 2: Figure S2). Seven of these motifs were found to be associated with the functionally defined domains. Motifs 1 and 2 were referred to the HD domain, which is the typical conserved domain found in the middle of all the TaHDZ proteins, and motif 5 was associated with the adjacent LZ domain. Motifs 17 and 34 were specifically made up the MEKHLA domain in subfamily III proteins of wheat (14 members). Motifs 3 and 4 were associated with the START region, which has been identified in subfamily III and IV proteins (Additional file 2: Figure S2). Similar motif compositions are shared by TaHDZ proteins which cluster together, and this indicates that members of a given group possess similar functionalities.

### Tissue-specific expression profile of *TaHDZ* genes

**Fig. 4:**
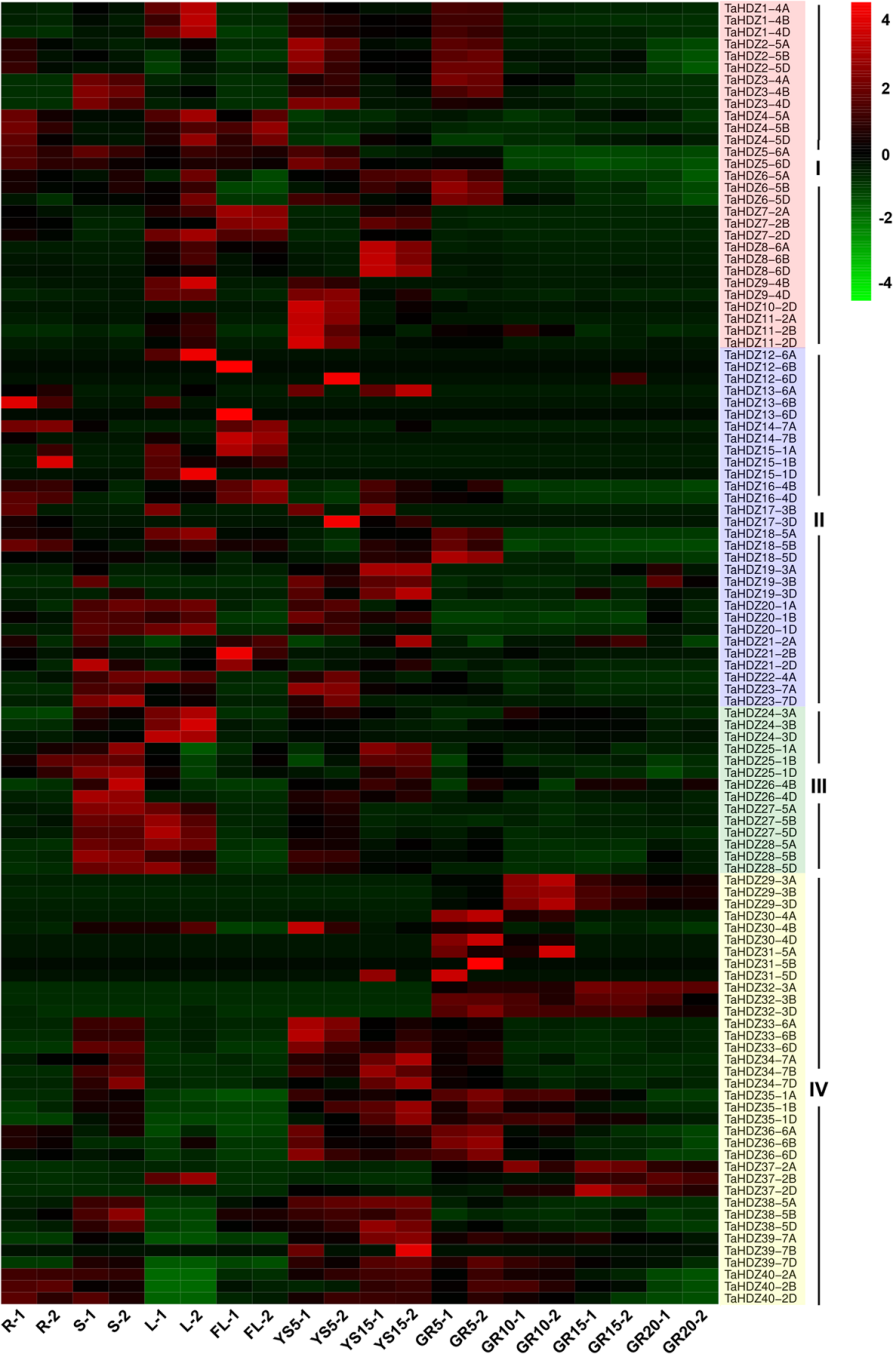
Expression profiles of *TaHDZ* genes in ten different organs or tissues. The heat map was drawn in Log_10_-transformed expression values. The red or green colors represent the higher or lower expression level of each transcript in each sample. R, root of wheat seedling at five-leaf stage; S, stem of wheat seedling at five-leaf stage; L, leaf of wheat seedling at five-leaf stage; FL, flag leaf at heading stage; YS5, young spike at early booting stage; YS15, spike at heading stage; GR5, grain of 5 days post-anthesis; GR10, grain of 10 days post-anthesis; GR15, grain of 15 days post-anthesis; GR20, grain of 20 days post-anthesis

Gene family members can exhibit different expression patterns in different tissues to accommodate various physiological processes. To gain insight into the temporal and spatial expression patterns and putative functions of *HD-Zip* genes in wheat growth and development, the tissue-specific expression patterns of the 113 *TaHDZ* genes were investigated using RNA-seq data from 10 different tissues. All *TaHDZ* genes were found to be expressed in at least one of the tissues examined (Fig. 4; Additional file 3: Table S1). Subfamily I *TaHDZ* genes were found to be much more highly expressed in seedling roots, stems, leaves, flag leaves, young spikes, and 5-day-old grains; for example, *TaHDZ1-4A/B/D* are highly expressed in leaves and 5-day-old grains, *TaHDZ8-6A/B/D* are highly expressed in leaves and young spikes (15-days-old), and *TaHDZ11-2A/B/D* are highly expressed in leaves and 5-day-old spikes (Fig. 4; Additional file 4: Figure S3). Subfamily II *TaHDZ* genes are more highly expressed in seedling roots, stems, leaves, flag leaves, and young spikes; for example, *TaHDZ19-3A/B/D* are highly expressed in young spikes, while *TaHDZ20-1A/B/D* are highly expressed in seedling stems, leaves, and 5-day-old spikes (Fig. 4; Additional file 5: Figure S4). Subfamily III *TaHDZ* genes showed relatively higher expression levels in seedling stems, leaves, and young spikes; *TaHDZ24-3A/B/D* are highly expressed in seedling leaves, and *TaHDZ27-5A/B/D* are highly expressed in seedling stems and leaves (Figure 4; Additional file 6: Figure S5). Subfamily IV *TaHDZ* genes are highly expressed in seedling stems, young spikes, and grains; *TaHDZ29-3A/B/D* are highly expressed in 10-day-old grains, *TaHDZ32-3A/B/D* are highly expressed in 5–20 day-old grains, and *TaHDZ38-5A/B/D* are highly expressed in seedling stems and young spikes (Fig. 4; Additional file 7: Figure S6). Thus, genes in the four wheat HD-Zip subfamilies display obvious differences in expression patterns and levels, which indicates that these genes have undergone functional differentiation and redundancy. It is worth mentioning that most homologous genes show similar expression patterns during development. However, it should also be noted that many clustered expression profiles do not reflect gene similarities, and this includes the copies of individual *HD-Zip* gene types from the sub-genomes. Some of them even show the opposite expression patterns. For instance, *TaHDZ7*, which is located on chromosome 2D, is preferentially expressed in the seedling leaves and flag leaves, whereas the homologous *TaHDZ7* gene from 2A is only expressed in the flag leaves, and the *TaHDZ7* homolog from 2B is preferentially expressed in flag leaves and 5-day-old spikes (Fig. 4; Additional file 4: Figure S3). *TaHDZ37* on 2A shows relatively higher expression in 10–15 day-old grains, while its homologous *TaHDZ37* from 2B is preferentially expressed in seedling leaves and 20-day-old grains, and the homologous from 2D is highly expressed in 15-days-old grains (Figure 4; Additional file 7: Figure S6). The divergences in expression profiles between homologous genes from the different subgenomes reveals that some of them may have lost their function or acquired a new function after polyploidization during the evolution of wheat.

### Expression patterns of *TaHDZ* genes in response to drought stress

**Fig. 5:**
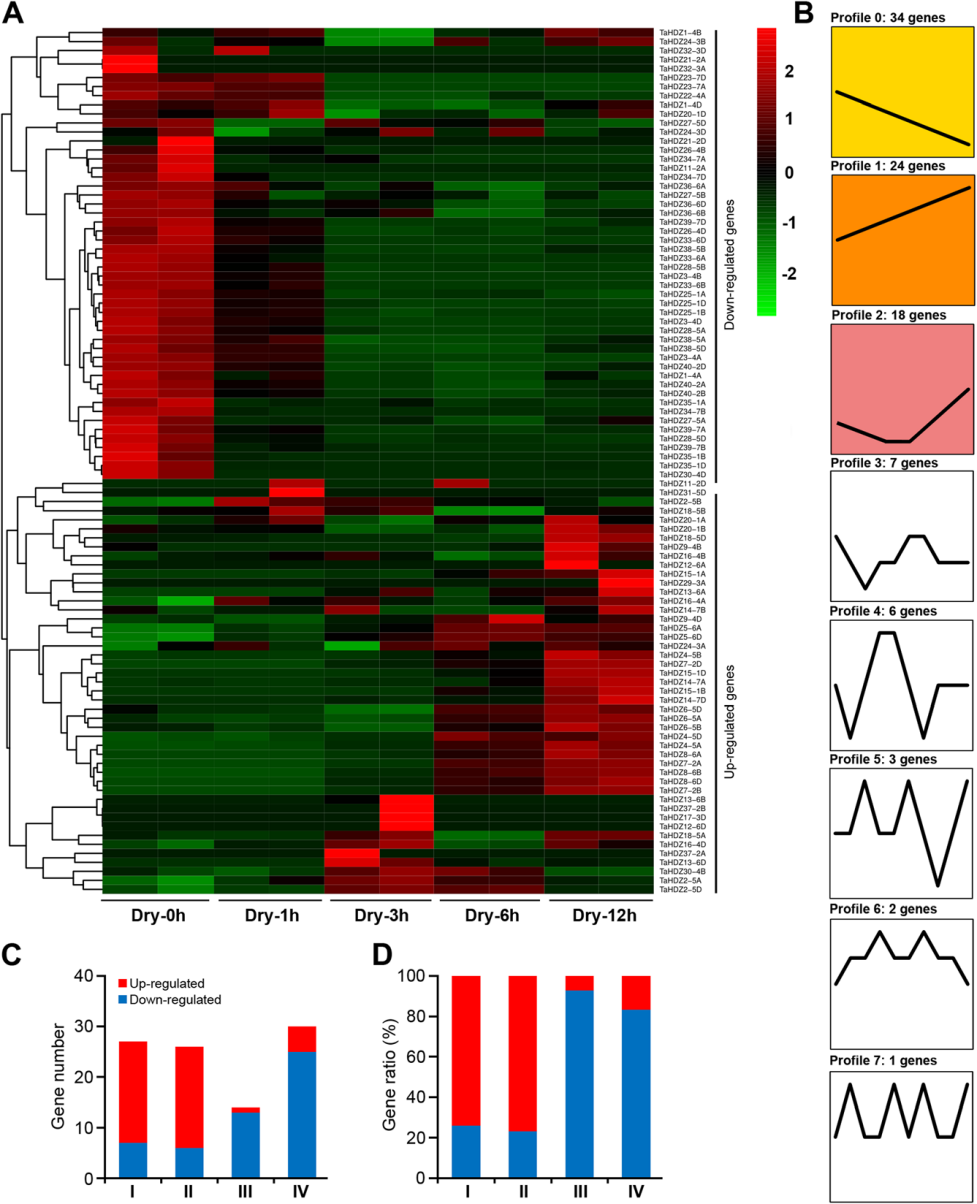
Expression profiles of *TaHDZ* genes in seedling leaves under drought stress treatment. **a** hierarchical clustering of the relative expression level of *TaHDZ* genes under drought stress treatment. The heat map was drawn in Log_10_-transformed expression values. The red or green colors represent the higher or lower relative abundance of each transcript in each sample. **b** Expression patterns of *TaHDZ* genes under drought stress treatment. **c** The numbers of up-regulated and down-regulated *TaHDZ* genes in four HD-Zip subfamilies. **d** The ratios of up-regulated and down-regulated *TaHDZ* genes in four HD-Zip subfamilies

**Fig. 6:**
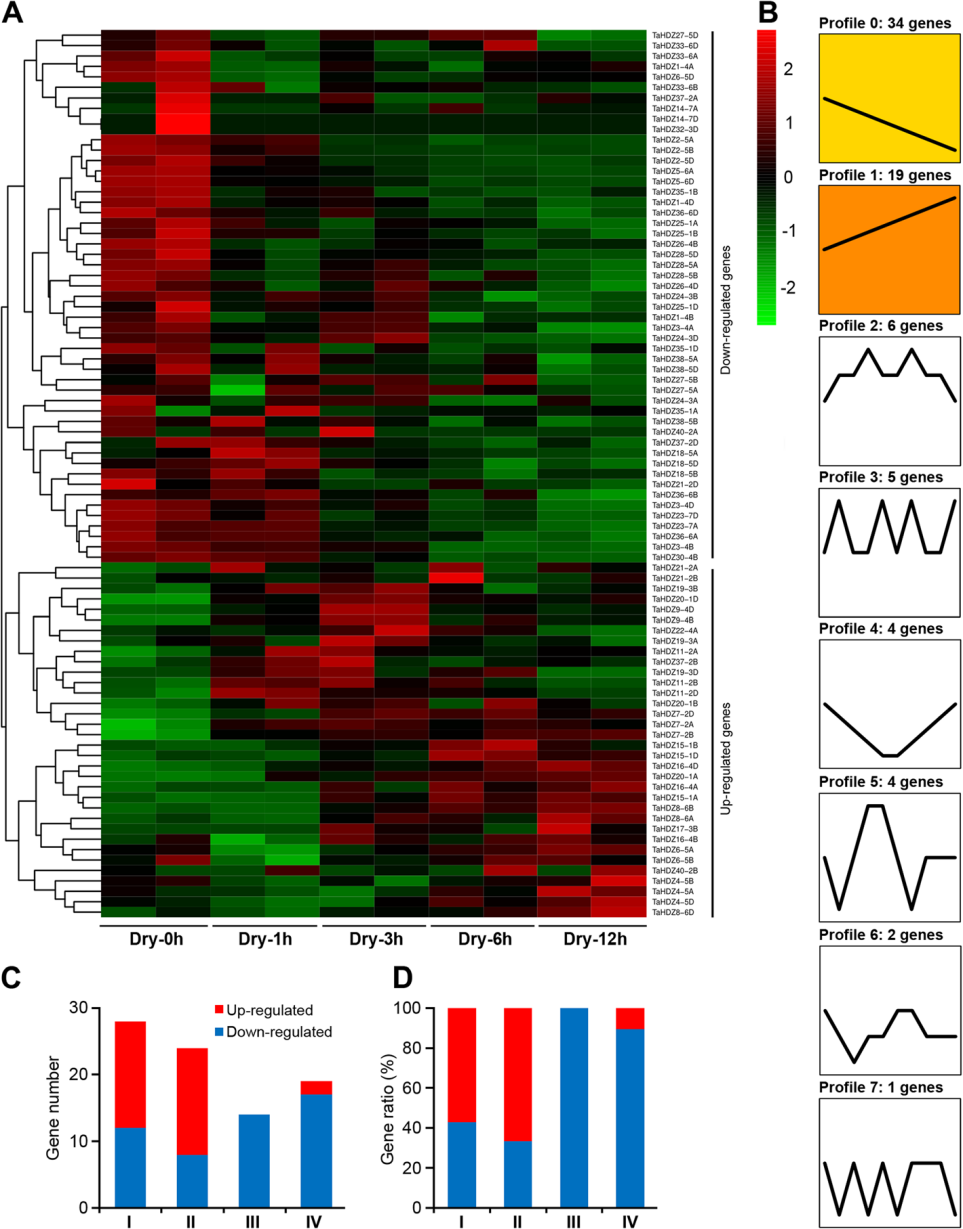
Expression profiles of *TaHDZ* genes in seedling roots under drought stress treatment. **a** Hierarchical clustering of the relative expression level of *TaHDZ* genes under drought stress treatment. The heat map was drawn in Log_10_-transformed expression values. The red or green colors represent the higher or lower relative abundance of each transcript in each sample. **b** Expression patterns of *TaHDZ* genes under drought stress treatment. **c** The numbers of up-regulated and down-regulated *TaHDZ* genes in four HD-Zip subfamilies. **d** The ratios of up-regulated and down-regulated *TaHDZ* genes in four HD-Zip subfamilies

Wheat productivity is severely affected by drought stress, and therefore the study of drought responsive genes is important to increase wheat yield. Many studies have shown that the *HD-Zip* genes play a crucial role in the response to abiotic stresses in plants. To gain more insight into the roles of wheat *HD-Zip* genes in stress tolerance, we first identified the *cis*-elements within 2 kb promoter region using online program PlantCARE (http://bioinformatics.psb.ugent.be/webtools/plantcare/html/). We found a number of *cis*-acting elements related to stress response in the promoter of *TaHDZ*s. They included DRE (Dehydration-responsive element), ABRE (ABA-responsive element), MBS (MYB binding site involved in drought-inducibility), MYC (MYC recognition site), MYB (MYB recognition site), and LTR (low temperature responsive element) (Additional file 8: Table S2). To further understand the potential role of *TaABFs* in the drought stress response, we reanalyzed the expression profiles of all wheat *HD-Zip* genes using RNA-seq data from roots and leaves that were subjected to drought treatment. We found that the wheat *HD-Zip* genes could be mainly classified into two groups based on their expression patterns (Fig. 5a, b; Fig. 6a, b). In leaves, the expression levels of 45 *TaHDZ* genes were up-regulated at one or more time point during drought stress treatment; this included 20 genes from the HD-Zip I subfamily (*TaHDZ2-5A/B/D*, *TaHDZ4-5A/B/D*, *TaHDZ5-6A/D*, *TaHDZ6-5A/B/D*, *TaHDZ7-2A/B/D*, *TaHDZ8-6A/B/D*, *TaHDZ9-4B/D*, and *TaHDZ11-2D*), 19 genes from the HD-Zip II subfamily (*TaHDZ18-5A/B/D*, *TaHDZ20-1A/B*, *TaHDZ16-4A/B/D*, *TaHDZ12-6A/D*, *TaHDZ13-6A/B/D*, *TaHDZ14-7A/B*, *TaHDZ15-1A/B/D*, and *TaHDZ17-3D*), one gene from the HD-Zip III subfamily (*TaHDZ24-3A*), and five genes from the HD-Zip IV subfamily (*TaHDZ29-3A*, *TaHDZ30-4B*, *TaHDZ31-5D*, *TaHDZ37-2A/B*) (Fig. 5a, c, and d). In contrast, 50 *TaHDZ* genes showed down-regulated expression under drought stress, including seven genes from subfamily I, six genes from subfamily II, 12 genes from subfamily III, and 25 genes from subfamily IV (Fig. 5a, c, d). In roots, 34 *TaHDZ* genes were found to be up-regulated in response to drought stress, including 16 genes from subfamily I (*TaHDZ4-5A/B/D*, *TaHDZ6-5A/B*, *TaHDZ7-2A/B/D*, *TaHDZ8-6A/B/D*, *TaHDZ9-4B/D*, and *TaHDZ11-2A/B/D*), 16 genes from subfamily II (*TaHDZ15-1A/B/D*, *TaHDZ16-4A/B/D*, *TaHDZ17-3B*, *TaHDZ19-3A/B/D*, *TaHDZ20-1A/B/D*, *TaHDZ21-2A/B*, and *TaHDZ22-4A*) and two genes from subfamily IV (*TaHDZ37-2B* and *TaHDZ40-2B*) (Fig. 6a, c, d). In contrast, 51 *TaHDZ* genes were down-regulated under drought stress in roots, including 12 genes from subfamily I, 8 genes from subfamily II, 13 genes from subfamily III, and 18 genes from subfamily IV (Fig. 6a, c, d). These results indicate that most *TaHDZ* genes in subfamilies I and II may play important roles in the response to drought stress.

### *TaHDZ5-6A* confers drought tolerance in *Arabidopsis*

**Fig. 7:**
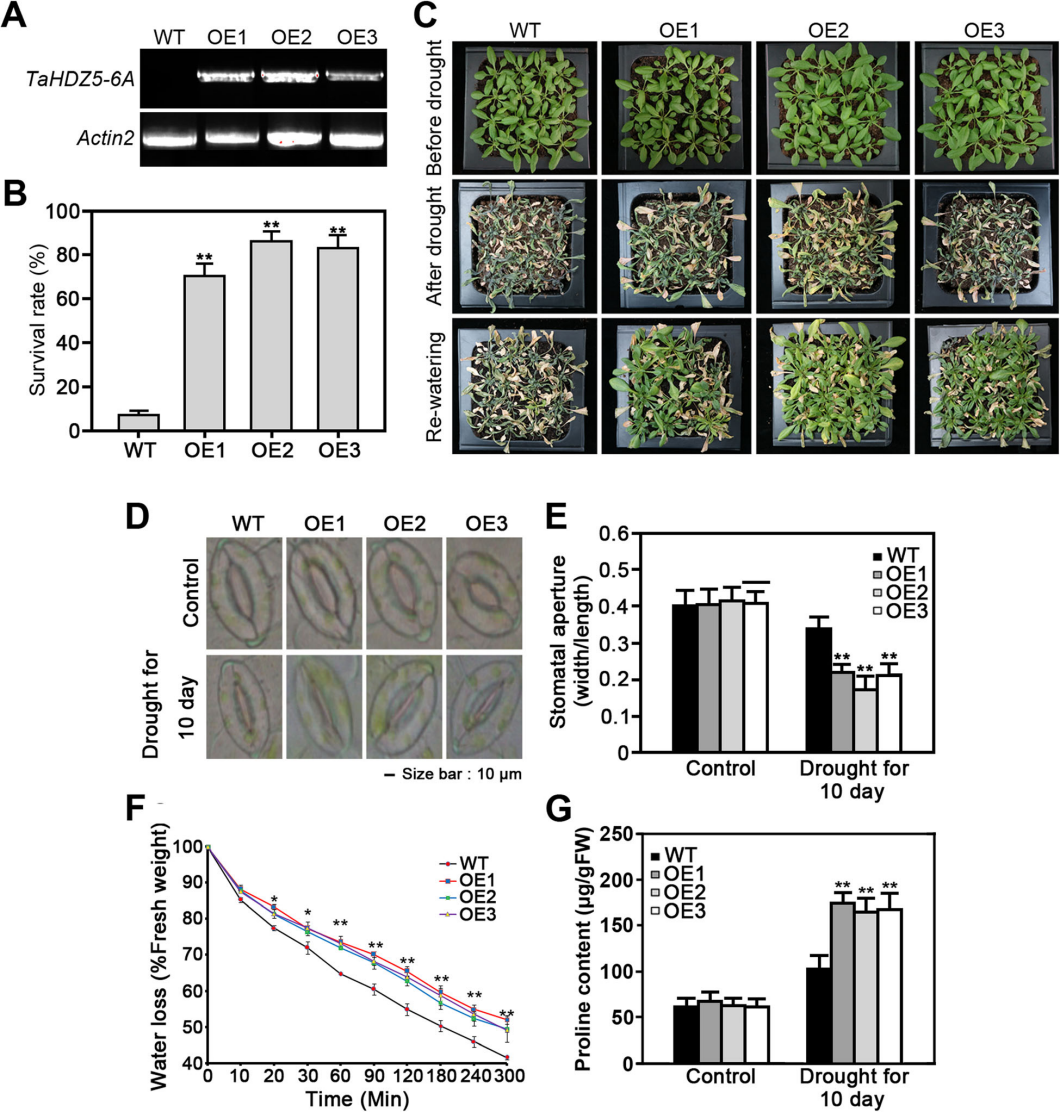
Phenotype of the *35S:TaHDZ5-6A* transgenic *Arabidopsis*. **a** RT-PCR analysis of *TaHDZ5-6A* transcript levels in the three transgenic lines. **b** Statistical analysis of survival rates after the drought-stress treatment. The average survival rates and standard errors were calculated based on data obtained from three independent experiments. Significant differences were determined by a *t*-test. *P < 0.05, **P < 0.01. **c** Drought tolerance of *35S:TaHDZ5-6A* transgenic *Arabidopsis*. Photographs were taken before and after the drought treatment, and followed by a six-day period of re-watering. **D** Stomatal aperture of WT and *35S::TaHDZ5-6A* transgenic plants under normal and drought conditions. **e** Statistical analysis of stomatal aperture of WT and *35S::TaHDZ5-6A* transgenic plants. Values are mean ratios of width to length. Error bars represent standard errors of three independent experiments (*n* = 60). Bars, 10 μm. **f** Water loss from detached rosettes of WT and *35S::TaHDZ5-6A* transgenic plants. Water loss was expressed as the percentage of initial fresh weight. Values are means from eight plants for each of three independent experiments. Significant differences were determined by a *t*-test. *P < 0.05, **P < 0.01. **g** Free proline content of WT and *35S::TaHDZ5-6A* transgenic plants under normal and drought stress treatment

The phylogenetic analysis and gene expression profiles suggest that *TaHDZ5-6A/D* may participate in regulating the drought stress response in wheat. Protein sequence analysis revealed that TaHDZ5-6A and TaHDZ5-6D share 95% sequence similarity (Additional file 9: Figure S7). In order to further comfirm the potential role of *TaHDZ5* in the drought stress response, we performed quantitative real-time PCR (qRT-PCR) using RNA isolated from different tissues and drought conditions. The PCR primers were designed to amplify the homologous alleles of *TaHDZ5*. The results showed that *TaHDZ5* is expressed at higher levels in the seedling leaves, flag leaves and young spikes, with the highest expression detected in the seedling leaves, and *TaHDZ5* was upregulated throughout the testing period by drought stress (Additional file 10: Figure S8). To further investigate the role of *TaHDZ5* in the drought stress response, we generated *35S::TaHDZ5-6A* transgenic *Arabidopsis* lines. Three independent transgenic lines (*OE1*, *OE2*, and *OE3*) were chosen for analysis based on their *TaHDZ5-6A* expression levels (Fig. 7a). WT and *35S::TaHDZ5-6A* transgenic plants were grown for 3 weeks in soil before water was withheld for 14 d. There was no obviously phenotypic differences between *35S::TaHDZ5-6A* transgenic and WT plants under normal conditions (Fig. 7c). After the drought treatment and six days of rewatering, 72–88% of the *35S::TaHDZ5-6A* plants had survived, whereas only ~ 8% of the WT plants were alive (Fig. 7b, c). Thus, the ectopic of *TaHDZ5-6A* greatly improved drought tolerance in transgenic *Arabidopsis*.

The stomatal apertures of leaves from *35S::TaHDZ5-6A* and WT plants grown in soil were measured. The stomatal aperture indices of of the *OE1*, *OE2*, and *OE3* plants were 0.41, 0.42 and 0.41, respectively, while that of the WT plants was 0.40, when grown under normal conditions (Fig. 7d, e). After being subjected to 10 d of drought stress, the stomatal aperture indices of the *OE1*, *OE2*, and *OE3* plants decreased to 0.22, 0.18, and 0.22, respectively, significantly reduced as compared to that of the WT (Fig. 7d, e). Consistent with these results, the water loss in detached leaves of *35S::TaHDZ5-6A* transgenic plants was much more slowly than those of WT plants under dehydration (Fig. 7f). These results indicate that the *35S::TaHDZ5-6A* transgenic plants removed water from the soil more slowly than did the WT plants, reducing the rate of wilting. To explore whether *TaHDZ5-6A* ectopic expression influences proline accumulation, we compared the free proline contents in *35S::TaHDZ5-6A* transgenic and WT plants. Consistent with the drought tolerance phenotype, the proline contents were much higher in transgenic plants than those of the WT plants under drought conditions (Fig. 7g). These findings collectively indicate that *TaHDZ5-6A* can enhance drought tolerance in transgenic *Arabidopsis*.

### Global gene expression changes in *35S::TaHDZ5-6A* transgenic *Arabidopsis*

**Fig. 8:**
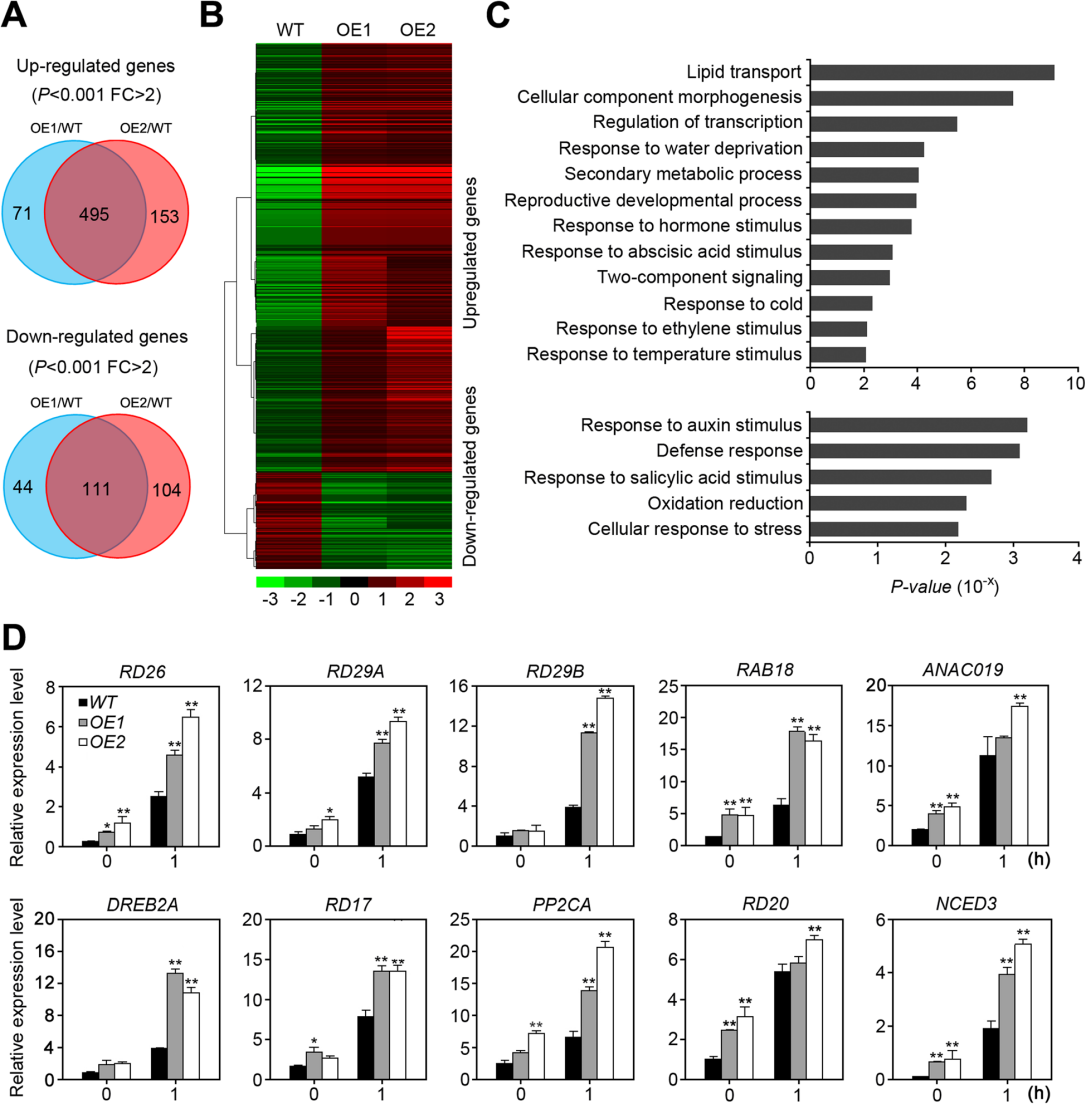
Transcriptomic analyses of the *35S::TaHDZ5-6A* transgenic *Arabidopsis* under normal condition. **a** venn diagrams of up- or down-regulated genes in transgenic plants relative to WT plants using a significance cutoff of *P* < 0.001, and a fold-change (FC) > 2. **b** Hierarchical clustering of up- or down-regulated genes in *35S::TaHDZ5-6A* transgenic *Arabidopsis* lines relative to WT plants. The indicated scale is the log_2_ value of the normalized level of gene expression. **c** Gene ontology of biological pathways (GOBPs) enriched in *TaHDZ5-6A* transgenic plants based on up or downregulated genes. **d** qRT-PCR analysis of drought induced genes in the transgenic and WT plants under normal and drought conditions. The error bars indicate standard deviations derived from three independent biological experiments. Significant differences were determined by a *t*-test. *P < 0.05, **P < 0.01

RNA sequencing allowed us to understand how drought tolerance was conferred by the ectopic of *TaHDZ5-6A*. The transcriptome of the *35S::TaHDZ5-6A* transgenic plants was compared to that of WT plants under normal, non-stress conditions. In transgenic plants, a total of 495 and 111 genes were upregulated and downregulated by at least 2-fold (*P* < 0.001, FDR < 0.05) as compared with the WT (Fig. 8a, b; Additional file 11: Table S3). The upregulated genes included genes related to water deprivation, abscisic acid, hormones, and abiotic stimuli, and downregulated pathways included those responsive to auxin stimuli, oxidative stress, and defense responses (Fig. 8c). We then chose 10 genes upregulated in transgenic plants and known to be involved in response to drought: *DREB2A* [39], *RD29A* [40], *RD29B* [40], *RD26* [41], *RD17* [42], *PP2CA* [43], *RAB18* [42], *ANAC019* [44], *NCED3* [45], and *RD20* [46]. We used qRT-PCR to measure their relative expression levels under normal and drought conditions in transgenic and WT plants (Fig. 8d). The results of qRT-PCR were in alignment with those of RNA-seq, indicating that *TaHDZ5-6A* may positively regulate the transcription of these 10 genes, and thereby play a role in the response, including rapid stomatal closure and reduction of water loss, of transgenic *Arabidopsis* plants under drought conditions.

## Discussion

Benefit from the whole genome sequencing and the genomic databases, we could explore the gene families in much greater detail, especially in complex genomes, such as wheat [47]. The identification of wheat *HD-Zip* genes is an essential step towards the further functional characterization of these genes. Although the *HD-Zip* gene family has been widely studied in both monocots and dicots, their functions remain obscure in wheat. Previous studies have reported the identification of a few individual *HD-Zip* gene families in wheat, including the *TaHDZipI-1* to *TaHDZipI-5* genes [10, 11, 34]. However, a systematic identification and characterization of wheat *HD-Zip* genes has not been performed until the present. To address this knowledge gap, we performed a comprehensive identification and analysis of wheat *HD-Zip* genes in this study.

We identified 113 putative *HD-Zip* genes in wheat based on the Chinese Spring IWGSC RefSeq v1.1 reference genome (https://wheat-urgi.versailles. inra.fr/) (Table 1). The number of *HD-Zip* genes is twice than that found in *Arabidopsis*, rice, maize, or poplar [3, 4, 37], because wheat is an allohexaploid crop. The further sequence alignment clearly showed the higher sequence divergence of 113 wheat HD-Zip proteins, especially the sequences at the C-terminus, indicating that *HD-Zip* genes play the diverse roles in wheat growth and development [2, 48]. The wheat HD-Zip family members can be further divided into four well-conserved subfamilies (I-IV) based on their phylogenic relationships (Fig. 1; Fig. 2), gene structures (Fig. 2), and motif arrangements (Additional file 2: Figure S2). Our results are consistent with previous studies [3, 4, 37]. The HD-Zip III was the smallest subfamily in our study (Fig. 1; Fig. 2), which is consistent with the earlier reports that HD-Zip III is the most conserved subfamily with little members among various species [3, 37]. Also, the number of HD-Zip II and IV subfamily members vary in different species, which is the main reason that there are different numbers of HD-Zip family genes in various species. The gene structure analysis revealed that genes in each HD-Zip subfamily have similar exon-intron structures with respect to numbers and positions (Fig. 3b). However, the HD-Zip II and HD-Zip IV subfamilies were found to be more divergent (Fig. 3c), indicating that these genes may have different functions in wheat development. In addition, wheat HD-Zip proteins contain specific conserved domains in each subfamily (Additional file 2: Figure S2). The HD and LZ domains, which have been reported to be responsible for protein-DNA and for protein-protein interactions, are conserved in all HD-Zip proteins [36]. Except for subfamily-biased conserved motifs, the HD-Zip proteins target different sequences; for example, the HD-Zip I proteins target the CAAT(A/T) ATTG sequence, HD-Zip II proteins interact with the CAAT(C/G) ATTG sequence, and HD-Zip III and IV proteins recognize GTAAT(G/C) ATTAC and TAAATG(C/T) A, respectively [10, 36].

Recently, there have been efforts to understand the functions of *HD-Zip* genes in *Arabidopsis*. To further elucidate the potential functions of wheat *HD-Zip* genes, the orthologous relationships between wheat and *Arabidopsis* proteins have been examined in depth. Subfamily I is divided into seven subclasses or clades, i.e., α, β, γ, δ, ε, φ, and ζ (Fig. 2). Clade α includes *Arabidopsis ATHB13*, a positive regulator of drought, salinity, and cold stresses [49, 50], that is a ortholog of the wheat *TaHDZ3-4A/B/D* genes. Expression of the β-clade members *ATHB5* and *ATHB6* is also affected by water deficit, and both genes appear to regulate growth in response to ABA and/or drought treatment [51, 52], but these genes have no orthologs in wheat. γ-Clade members are typically induced by abiotic stresses, and include *Arabidopsis ATHB7* and *ATHB12* [7], which are the orthologs of the wheat *TaHDZ5-6A/B/D*, *TaHDZ7-2A/B/D*, and *TaHDZ8-6A/B/D* genes. Furthermore, the δ-clade genes *ATHB21*, *ATHB40*, and *ATHB53* are induced by ABA treatment and salt stress; these three TFs are involved in controlling axillary bud development [53], and are the orthologs of the wheat *TaHDZ9-4A/B/D*, *TaHDZ10-2B/D*, and *TaHDZ11-2A/B/D* genes. The HD-Zip II subfamily is divided into ten subclasses, from α to κ (Fig. 2). Clade γ consists of *ATHB2/HAT4* and *HAT2*, genes that regulate auxin-mediated morphogenesis in *Arabidopsis* [13, 14] and that are the orthologs to wheat *TaHDZ21-2A/B/D*. The HD-Zip III subfamily is classified into three subclades, designated α, β, and γ (Fig. 2). Clade α corresponds to the *REV* clade described in previous studies [54], which are orthologs of the wheat *TaHDZ25-1A/B/D* and *TaHDZ26-4B/D* genes. Clade β includes *Arabidopsis ATHB8* [17] and *ATHB15/CNA* [19], which are the orthologs of wheat *TaHDZ24-3A/B/D*, and clade γ contains PHB and PHV [55, 56], which are the orthologs of wheat *TaHDZ27-5A/B/D* and *TaHDZ28-5A/B/D*. The HD-Zip IV subfamily also consists of six subclades, designated α, β, γ, δ, ε, and ζ (Fig. 2). Clade α contains *Arabidopsis ANL2*, a regulator of anthocyanin accumulation in the leaf sub-epidermal layer and of cell identity in the root [57]; *ANL2* is orthologous to the wheat *TaHDZ36-6A/B/D*, *TaHDZ37-2A/B/D*, and *TaHDZ38-5A/B/D* genes. Clade β includes *Arabidopsis GL2* [22], which is the ortholog of wheat *TaHDZ27-5A/B/D* and *TaHDZ32-3A/B/D*. Clade γ contains trichome formation genes, *HDG4*, *HDG5*, and *HDG8–12* [58], which are the orthologs of wheat *TaHDZ33-6A/B/D*, *TaHDZ34-7A/B/D*, and *TaHDZ35-1A/B/D*. Clade ε is composed of *AtML1* and *PDF2* that are responsible for shoot epidermal cell differentiation [21], and are the orthologs of the wheat *TaHDZ39-7A/B/D* genes. These results will help us to further understand the function of wheat *HD-Zip* genes, especially those that are orthologous with *Arabidopsis HD-Zip* genes.

To better understand the roles of the wheat *HD-Zip* genes during the life cycle of wheat, we performed an expression analysis of publicly-available RNA-seq data in 10 organs/tissues at different developmental stages. Genes in the HD-Zip family have been reported to be involved in the development of different organs, and expression *HD-Zip* genes varies widely in different organs (Fig. 4); for example, Genes of the HD-Zip I subfamily play an important role in the development of flowers and leaves, and have been found to control the development of cotyledon, spike, and leaves [8, 10, 59, 60]. We found that the HD-Zip I genes were primarily found in seedling leaves, flag leaves, and young spikes (Additional file 4: Figure S3). We also found that most of the HS-Zip II genes displayed elevated levels of expression in both leaves and young spikes (Additional file 5: Figure S4), while previous research had found that HD-Zip II genes were involved in carpel margin, flower growth [15, 61] and leaf polarity [62]. We also observed that HD-Zip III genes were found primarily in the leaves and stems of seedlings (Additional file 5: Figure S5), and might be involved in organ polarity, vascular development, and meristem function [54, 63]. Prior research has found that HD-Zip IV genes serve a role in the development of grain, trichome, and anther [23, 64], because most of them show higher expression levels in seedling stems, young spikes, and during grain development (Additional file 7: Figure S6). These results suggest the *TaHDZ* genes may play a variety of roles in wheat development.

The manner in which *HD-Zip* genes respond to stress strongly indicates that they are involved in adapting to dynamic conditions in their environment. The expression of the HD-Zip family I and II genes is activated or inhibited by drought conditions, which is similar to other plants (Fig. 5; Fig. 6). Our qRT-PCR analyses further revealed that a noval HD-Zip I gene, *TaHDZ5* is highly expressed in seedling leaves and is induced by drought stress (Additional file 10: Figure S8). To investigate the role of *TaHDZ5* in the abiotic stress response, We transformed the homologous gene *TaHDZ5-6A* into *Arabidopsis*, confirming the expression of *TaHDZ5-6A* via qRT-PCR (Fig. 7a). Compared to the WT plants, the transgenic lines were significantly more resistant to drought conditions. Environmental stressors can induce physiological changes in plants, which can be measured and used to analyze certain crops’ resistance to abiotic stressors. We analyzed the transpiration rate of the detached leaves, and found that the rate of water loss was lower in the transgenic plants than it was in the WT plants (Fig. 7f). We also found that under the stress of drought, the stomata closed at a quicker rate in the transgenic plants than it did in the WT plants (Fig. 7d, e). In addition, the proline content was higher in the transgenic plants compared to WT plants under drought conditions (Fig. 7g). We also found that constitutive expression of *TaHDZ5-6A* in *Arabidopsis* significantly increased the transcription of many stress-responsive genes, including *RD29A*, *RD29B*, *RAB18*, *DREB2A*, *NCED3*, and *RD17* (Fig. 8). These data provide strong evidence that *TaHDZ5-6A* can enhance drought tolerance in the transgenic arabidopsis plants. Previous studies have reported that overexpression of TF genes may cause the growth retardation in transgenic plants [65–67], restricting the applicability of target genes in transgenic breeding. However, in our study, there are no obvious adverse effects were observed of *35S::TaHDZ5-6A* transgenic plants (Fig. 7a), indicating the potential for using *TaHDZ5-6A* in plant breeding.

## Conclusions

In conclusion, we performed a comprehensive analysis of the genome organization, evolutionary relationships, and expression profiles of the HD-Zip gene family members in wheat and also functionally characterized *TaHDZ5-6A* by showing that it confers drought tolerance in transgenic *Arabidopsis*. The present study has built a foundation and provides an essential framework for the further functional characterization of wheat *HD-Zip* genes in various physiological processes, including their role and the underlying molecular mechanism in the regulation of drought tolerance in wheat.

## Methods

### Plant materials and drought stress treatments

Wheat (*Triticum aestivum*) variety Chinese spring (CS) was identified and obtained from the Prof. Zhensheng Kang’s Lab (Northwest A&F University, China) and was used to analysis the expression of *TaHDZ* genes, this wheat variety can also obtained from Chinese Crop Germplasm Resources Information System (http://www.cgris.net/ zhongzhidinggou/index.php). After surface-sterilized with 75% ethanol and washed with deionized water, the seeds were placed on wet filter paper to germinated at 25 °C for 3 days. The germinated seeds were placed in a nutrient solution (0.1 mM KCl, 0.75 mM K_2_SO_4_, 0.65 mM MgSO_4_, 0.25 mM KH_2_PO_4_, 1.0 mM MnSO_4_, 1.0 mM ZnSO_4_, 0.1 mM EDTA-Fe, 2.0 mM Ca (NO_3_)_2_, 0.005 mM (NH_4_)_6_Mo_7_O_24_, 0.1 mM CuSO_4_) for hydroponic cultivation with a 16/8 h light/dark cycle at 16 °C in a growth chamber. For drought treatment, the three-leaf stage seedlings were placed on a clean bench and subjected to dehydration (25 °C, relative humidity of 40–60%). Leaves and roots from three seedlings were collected after 0, 1, 3, 6, 12 and 24 h for drought treatment.

To investigate the tissue-specific expression patterns of *TaHDZ5-6A* in wheat, field grown wheat cv. Chinese spring were used. Wheat plants were grown during the growing season at the experimental station of the Northwest A & F University, Yangling, Shanxi, China (longitude 108°E, latitude 34°15′N) from 2016 to 2017. Ten tissue/organ samples including root, stem, leaf of wheat seedling at five-leaf stage, young spike at early booting stage, spike at heading stage, flag leaf at heading stage, and the grain of 5, 10, 15 and 20 DPA. Each sample was collected from at least five individual plants for two repeats. The aforementioned samples were frozen quickly in liquid nitrogen and placed at − 80 °C for further RNA extraction.

### Identification and annotation of *HD-Zip* genes in wheat

The HD-Zip domain (PF00046) was downloaded from Pfam (http://pfam.xfam.org/) and used as a query. The *HD-Zip* genes were identified from the Chinese Spring IWGSC RefSeq v1.0 reference genome assembly (https://wheat-urgi.versailles.inra.fr/) using HMMER3.1 [68](*E*-value <1e-10). After remove all redundant sequences using CD-hit program, the rest of protein sequences were further subjected to identify the HD and LZ domains using the Simple Modular Architecture Research Tool (SMART; http://smart.embl-heidelberg.de/smart/set_mode.cgi? NORMAL = 1). We further filtered these genes through phylogenetic analysis along with previously identified HD-Zip proteins from *Arabidopsis thaliana*, *Vitis vinifera*, *Populus trichocarpa*, *Brachypodium distachyon*, *Oryza sativa*, *Zea mays*, and *Physcomitrella patens* [37]. Phylogenetic analysis was also implemented to categorize different HD-Zip subfamilies. Homeologous genes from each of the three wheat subgenomes (A, B, and D genomes) were named *TaHDZX_ZA*, *TaHDZX_ZB*, or *TaHDZX_ZD*, where X denotes the gene number and Z the wheat chromosome where it is located. The theoretical pI (isoelectric point) and Mw (molecular weight) of each putative wheat HD-Zip protein was calculated using compute pI/Mw tool online (http://web.expasy. org/compute_pi/).

### Phylogenetic analysis and conserved protein motif/domain identification

Multiple sequence alignments were generated using the ClustalW program with the default settings [69]. To investigate the evolutionary relationship among HD-Zip proteins, an unrooted phylogenetic tree was obtained by neighbor-joining (NJ) method using MEGA6.0 software based on the full-length of HD-Zip protein sequences [70]. The bootstrap probability of each branch was estimated with 10,000 replications to obtain confidence support.

The gene structure imformation of *TaHDZ* genes were got from the Chinese Spring IWGSC RefSeq v1.0 reference genome, and analysed using the Gene Structure Display Server 2.0 (GSDS; http://gsds.cbi.pku.edu.cn/). The conserved motifs of TaHDZs were identified using SALAD database (http://salad.dna.affrc.go.jp/salad/ en/).

### Gene expression analysis by RNA-seq data

To study the expression of *TaHDZ* genes in different tissues, RNA-seq data from ten tissues, including root, stem, leaf of wheat seedling at five-leaf stage, young spike at early booting stage, spike at heading stage, flag leaf at heading stage, and the grain of 5, 10, 15 and 20 DPA were collected from database (http://genedenovoweb.ticp.net: 81/Wheat_GDR1246/index.php?m = index&f = index). For further analysis the expression of *TaHDZ* genes in response to drought stress, we harvested the leaves and roots from three-week-old wheat seedlings subjicted to drought treatment at 0, 1, 3, 6, and 12 h to conduct the RNA-seq analysis. Wheat plantation and sampling was mentioned above. TopHat and Cufflinks were used to analyze the genes’ expression based on the RNA-seq data [71, 72]. The FPKM value (fragments per kilobase of transcript per million fragments mapped) was calculated for each *TaHDZ* gene, the log10-transformed (FPKM + 1) values of the 113 *TaHDZ* genes were used for heat map generation.

### RNA extraction and quantitative real-time PCR

Total RNA was isolated and purified using Total RNA Rapid Extraction Kit for Polysaccharides Polyphenol Plant (BioTeke) according to the manufacturer’s directions. To eliminate genomic DNA contamination, the purified RNA was dosed with RNase-free DNase I (TaKaRa, China). One μg of the total RNA was used to synthesize first-strand cDNA via Recombinant M-MLV reverse transcriptase (Promega, USA). We then performed quantitative real time-PCR (qRT-PCR) using an ABI7300 Thermo-cycler (Applied Biosystems, USA) in optical 96-well plates. The reactions were performed in a 10 μl solution (200 nM gene-specific primers, 1 μl diluted cDNA, and 5 μl SYBR Premix Ex Taq II (TaKaRa)) under the following conditions: 10 min at 95 °C, then 40 cycles of 15 s at 95 °C and 30 s at 60 °C. We used a melting curve analysis to verify the specificity of the amplicon for each primer pair. The wheat *Actin* (Gene ID: 542814) was used as the internal control to detect the expression of *TaHDZ5* in wheat, and *Arabidopsis Actin2* (AT3G18780) was used to analyze the expression of stress-responsive genes in *Arabidopsis*. The relative gene expression levels were calculated according to the 2^−ΔΔCt^ method [73], with the variation in expression being estimated from three biological replicates. The primer pairs used for qRT-PCR analysis are listed in Additional file 12: Table S4.

### *TaHDZ5-6A* isolation and *Arabidopsis* transformation

*Arabidopsis* ecotype Columbia was obtained from the Prof. Zhensheng Kang’s Lab (Northwest A&F University, China) and was used to transform the *TaHDZ5-6A*. We amplified the full-length opening reading frame of *TaHDZ5-6A* with gene specific primers from wheat cDNA (forward: 5′-ATGGAGCCCGGCCGGCTCAT-3′; reverse: 5′ -CTAGTTCCACATCCAGTAGCTGATC-3′), after which we cloned into the pGreen vector [74] via the cauliflower mosaic virus (CaMV) 35S promoter. We then introduced the recombinant vector (*35S::TaHDZ5-6A*) into *Agrobacterium tumefaciens*, creating the ecotype Columbia via the floral dip method. We plated T_1_ seeds on an MS medium (2% sucrose, 50 mg/mL kanamycin) to select the transformants, and used homozygous T_3_ plants to analyze the phenotypes.

### Drought tolerance assay

We transferred seven-day-old *35S::TaHDZ5-6A* plants that were germinated on the MS medium to pots with a 230 g, 2:1 solution of Jiffy mix and vermiculite to perform the drought tolerance assays. We exposed 30 two-day-old plants that had been growing under ideal conditions (relative humidity 60%, and 16/8 h light/dark photoperiod, 22 °C) to drought conditions, withholding water from the plants for 14 days. We then resumed watering to allow for recovery, observing the number of plants that survived after six days. We compared at least 64 plants in each line with the wild-type (WT) plants in each experiment. The statistical data was obtained from three independent experiments, while a student’s *t*-test was used to analyze the differences between transgenic and wild-type plants.

### Water loss measurement

We measured the rates of water loss in eight plants of the *35S::TaHDZ5-6A* transgenic and eight wild-type plants. We detached soil-grown plants that were three weeks old from the roots, and weighed them immediately (fresh weight, FW). The plants were placed on a stable surface (relative humidity 40–45%, 22–24 °C) and weighed at predetermined intervals (desiccated weights), after which the proportions of weight loss were calculated relative to the starting weights. The plants were then dried in an oven at 80 °C for 24 h to a constant dry weight (DW). The water loss was considered to be the percentage of the starting weight at each predetermined time point. Each line had three replicates performed, and a student’s *t*-test was used to analyze the differences between transgenic plants and wild-type plants.

### Stomatal aperture analysis

Stomatal apertures were measured according to previously described procedures [75]. Samples of similarly sized and aged leaves were obtained from *35S::TaHDZ5-6A* and WT plants that were subjected to drought conditions for 10 d. The rosette leaves were placed in a solution (10 mM Mes-Tris, 30 mM KCl, pH 6.15) and exposed to light for 3 h. The stomata on the epidermal strips obtained from rosette leaves was examined using a light microscope (Olympus ix71, Tokyo, Japan). The Image J software (http://rsbweb.nih.gov/ij) was used to determine the length and width of the stomatal pores, which was subsequently used to calculate the stomatal apertures, or the ratio of width to length.

### Proline content measurement

After exposure to drought conditions, the *Arabidopsis* leaves were collected at predetermined times to assess the levels of free proline. We obtained leaves of similar size and location from WT plants and *35S::TaHDZ5-6A* to keep the samples uniform, and levels of free proline were analyzed according to previous procedures [76]. The samples (~ 0.1 g) were then homogenized in 3% sulfosalicylic acid and boiled for 10 min. Following the reaction between acid ninhydrin and proline, we measured the absorbance of the sample solutions at 520 nm using a UV-Vis spectrophotometer (NanoDrop 2000c, Thermo Scientific, Wilmington, DE, USA).

### RNA-seq analysis

Three-week-old *Arabidopsis* seedlings were harvested from *35S::TaHDZ5-6A* transgenic and WT plants under normal conditions to perform the RNA-seq analysis. The total RNA was isolated using Total RNA Rapid Extraction Kit for Polysaccharides Polyphenol Plant (BioTeke) according to the manufacturer’s directions. The preliminary quantitative of the concentration and purity of the total RNA were implemented using NanoDrop 2000 spectrophotometer (Thermo) and RNase free agarose gel electrophoresis. To remove the residual DNA, the extracted RNA was treated with RNase-free DNase I (New England Biolabs) for 30 min at 37 °C. Libraries from the resulting total RNA were prepared using the TruSeq paired-end mRNA-Seq kit and followed by multiplex adapter ligation, and 125 base paired-end sequencing on the Illumina Hiseq-2500 platform. Differential gene expression was determined using Tuxedo RNA-seq analysis pipeline [71, 72]. The DAVID software program was used to perform enrichment analyses of the gene ontology of biological pathways (GOBPs) [77], which calculated *P*-values to understand the significance of each GOBP that was represented by the genes. GOBPs with *P* < 0.01 were considered enriched processes.

## Supplementary information

**Additional file 1: Figure S1.** Distribution of *TaHDZ* genes among 21 chromosomes of wheat genome.

**Additional file 2: Figure S2.** Schematic representation of the conserved motifs in the TaHDZ proteins. Each motif is represented by a colored box. The black lines represent the non-conserved sequences.

**Additional file 3: Table S1.** FPKM (Fragments Per Kilobase Million) values of the *TaHDZ* genes in ten tissues and under drought stress treatment.

**Additional file 4: Figure S3.** Hierarchical clustering of the relative expression level of family I *TaHDZ* genes in ten different organs or tissues. The heat map was drawn in Log_10_-transformed expression values. The red or green colors represent the higher or lower expression level of each transcript in each sample.

**Additional file 5: Figure S4.** Hierarchical clustering of the relative expression level of family II *TaHDZ* genes in ten different organs or tissues. The heat map was drawn in Log_10_-transformed expression values. The red or green colors represent the higher or lower expression level of each transcript in each sample.

**Additional file 6: Figure S5.** Hierarchical clustering of the relative expression level of family III *TaHDZ* genes in ten different organs or tissues. The heat map was drawn in Log_10_-transformed expression values. The red or green colors represent the higher or lower expression level of each transcript in each sample.

**Additional file 7: Figure S6.** Hierarchical clustering of the relative expression level of family IV *TaHDZ* genes in ten different organs or tissues. The heat map was drawn in Log_10_-transformed expression values. The red or green colors represent the higher or lower expression level of each transcript in each sample.

**Additional file 8: Table S2.** Potential *cis*-elements within a 2 kb region upstream from the start codon of each *TaHDZ* gene.

**Additional file 9: Figure S7.** Protein sequence alignment of TaHDZ5-6A and TaHDZ5-6D. Identical amino acids are shaded in black, and similar amino acids are shaded in gray.

**Additional file 10: Figure S8.** The expression patterns of *TaHDZ5* in wheat. A The expression profiles of *TaHDZ5* in different tissues. R, root of wheat seedling at five-leaf stage; S, stem of wheat seedling at five-leaf stage; L, leaf of wheat seedling at five-leaf stage; FL, flag leaf at heading stage; YS5, young spike at early booting stage; YS15, spike at heading stage; GR5, grain of 5 days post-anthesis; GR15, grain of 15 days post-anthesis. B The expression pattern of *TaHDZ5* under drought stress treatment. The error bars indicate standard deviations derived from three independent biological experiments.

**Additional file 11: Table S3.** Genes up or downregulated in *35S:TaHDZ5-6A* transgenic *Arabidopsis* relative to WT plants under well-watered condition. Genes with an average fold change (FC) > 2.0 and a corrected-*P* < 0.001 are shown. The gene functional description is based on TAIR 10.

**Additional file 12: Table S4.** Primers used in this research. The name of the primers was based on the gene name and experimental purpose.

## Data Availability

The relevant data sets supporting the results of this article are included within the article and its additional files.

## Abbreviations

ABA: Abscisic acid
BLAST: Basic local alignment search tool
CS: Chinese spring
DW: Dry weight
FDR: False discovery rate
FPKM: Fragments per kilobase of transcript per million fragments mapped
FW: Fresh weight
GOBP: Gene ontology of biological pathway
HD: Homeobox domain
HD-SAD: HD-START-associated domain
LZ: Leucine zipper motif
Mw: Molecular weight
NJ: Neighbor-joining
OCL4: Outer cell layer4
pI: Isoelectric point
PRE: PopREVOLUTA
qRT-PCR: Quantitative reverse transcription polymerase chain reaction
START: Steroidogenic acute regulatory protein-related lipid transfer
TF: Transcription factor
WT: Wild-type
