# Supplementary figures and images for: Characterization of wheat homeodomain-leucine zipper family genes and functional analysis of TaHDZ5-6A in drought tolerance in transgenic Arabidopsis
Source: BMC Plant Biol. 2020 Jan 31;20:50. doi: 10.1186/s12870-020-2252-6 (PMC6993422; doi:10.1186/s12870-020-2252-6)

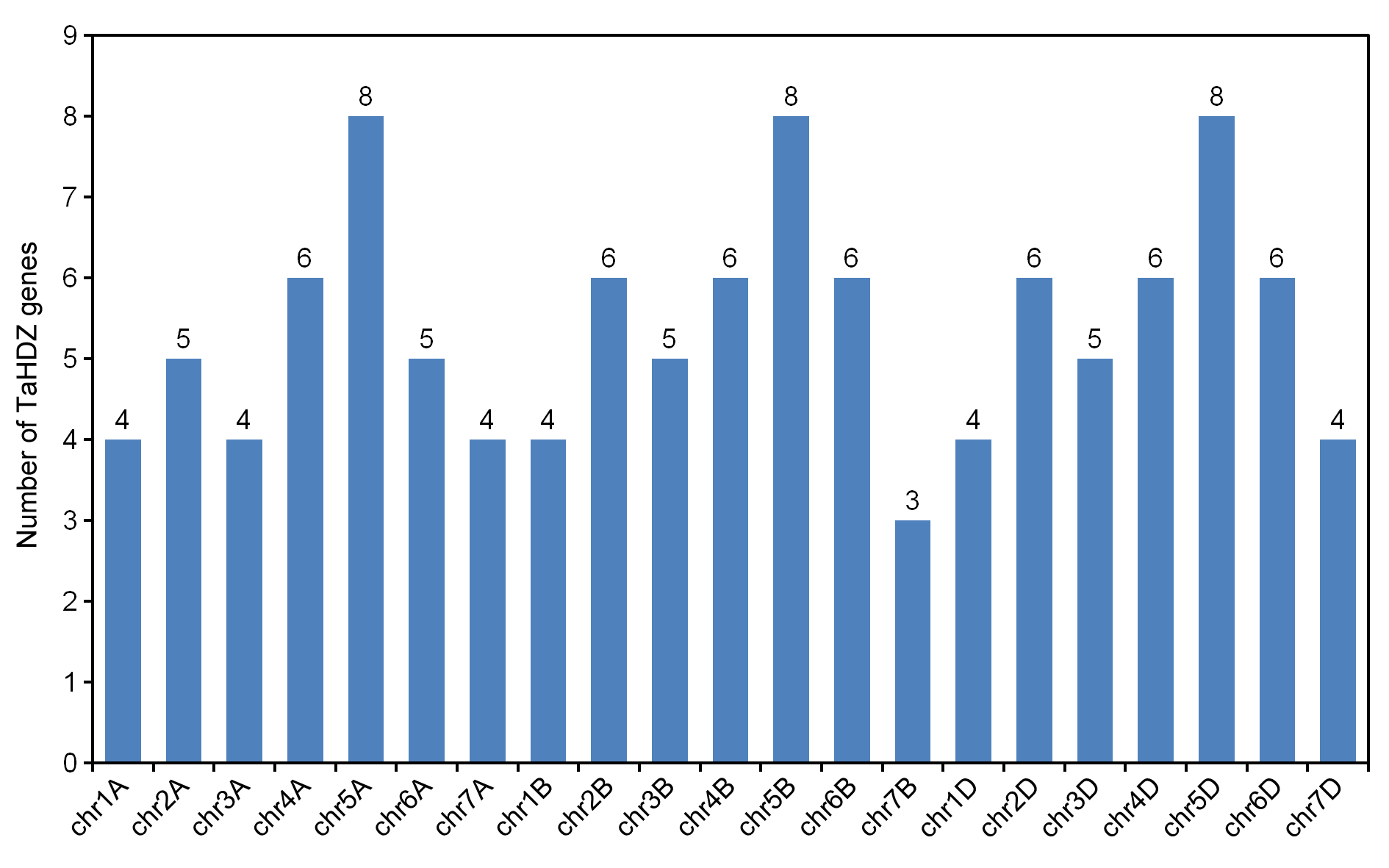

Supplement: Supplementary file 1 — Additional file 1: Figure S1. Distribution of TaHDZ genes among 21 chromosomes of wheat genome. [file 12870_2020_2252_MOESM1_ESM.tif]

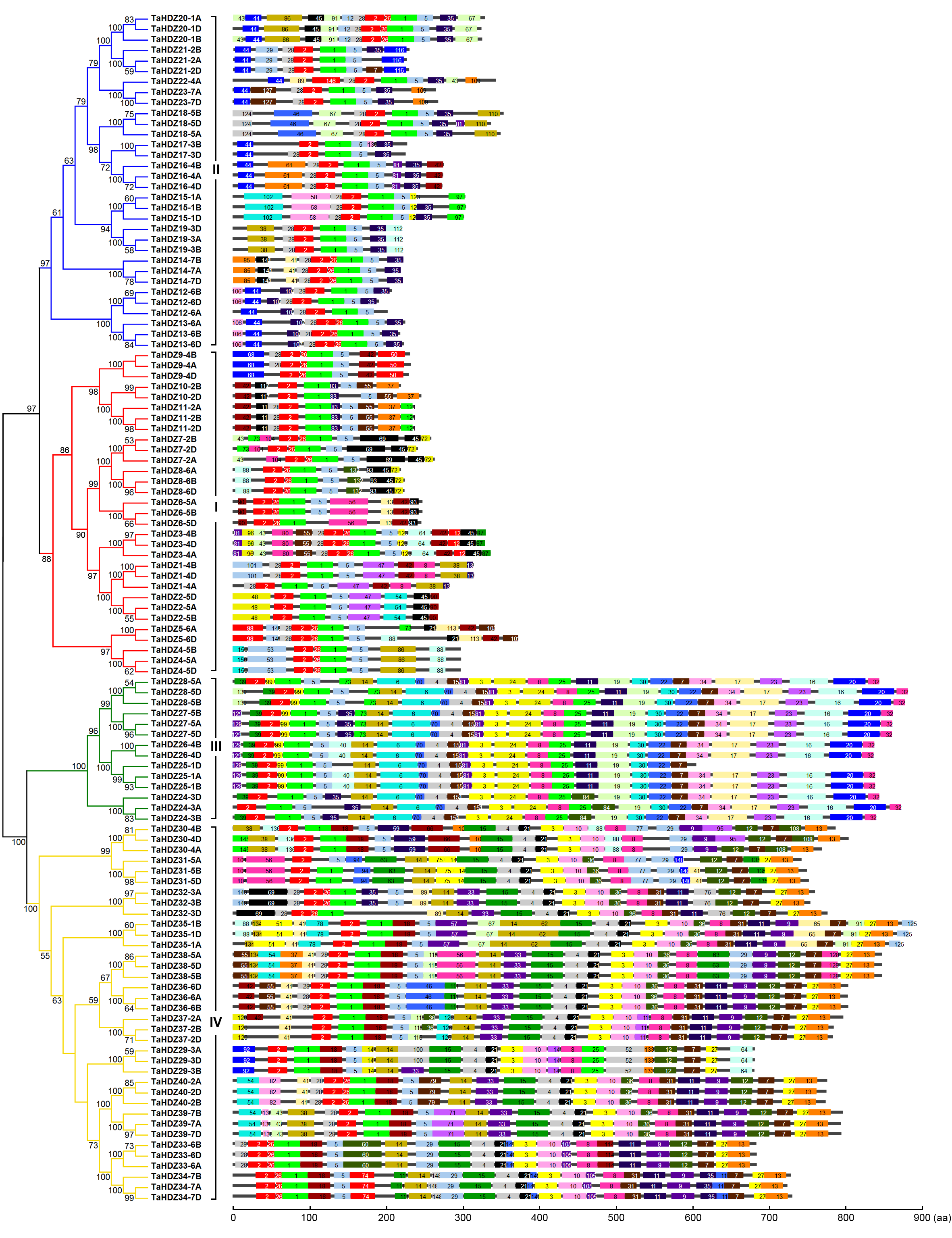

Supplement: Supplementary file 2 — Additional file 2: Figure S2. Schematic representation of the conserved motifs in the TaHDZ proteins. Each motif is represented by a colored box. The black lines represent the non-conserved sequences. [file 12870_2020_2252_MOESM2_ESM.tif]

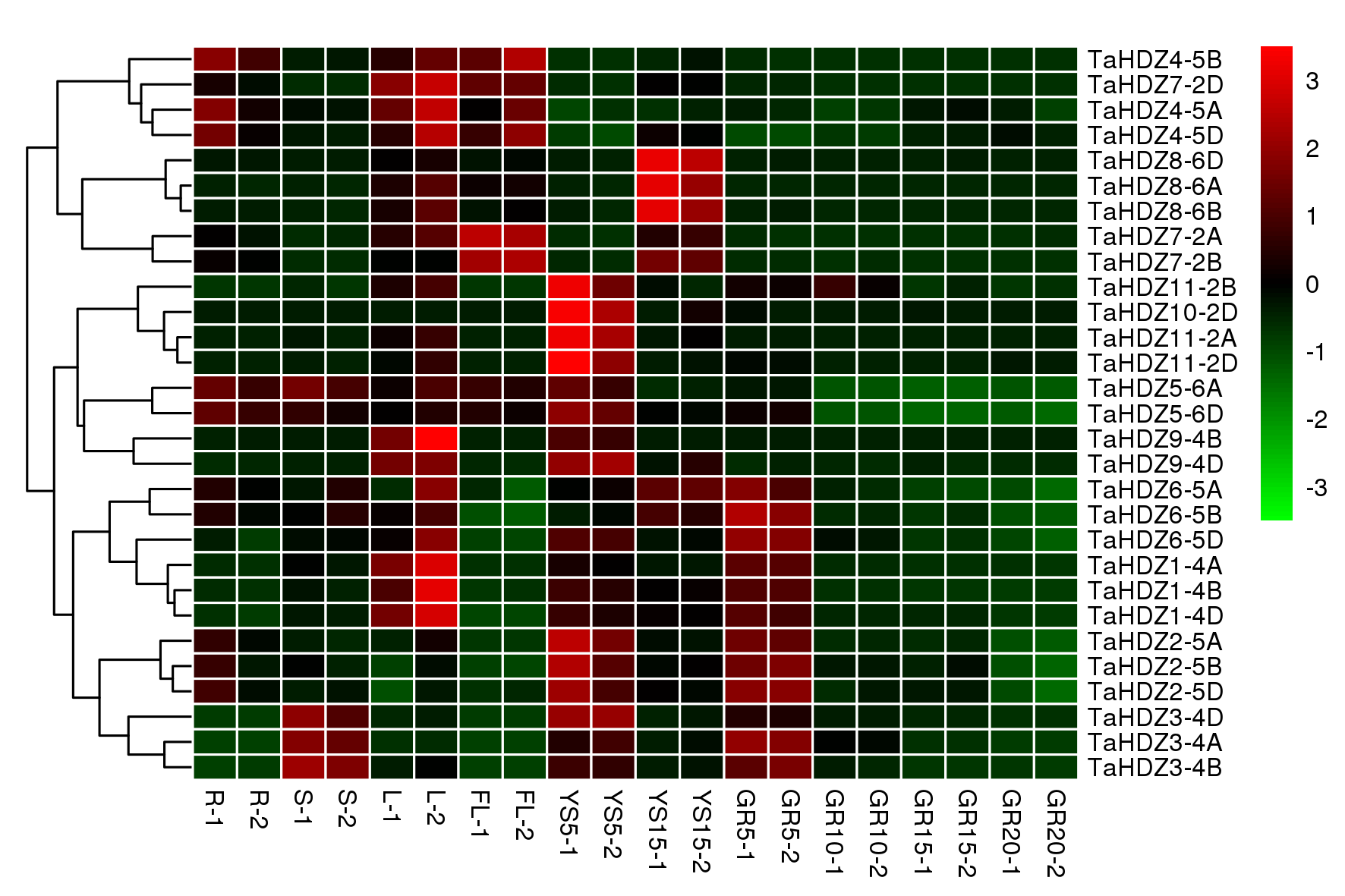

Supplement: Supplementary file 4 — Additional file 4: Figure S3. Hierarchical clustering of the relative expression level of family I TaHDZ genes in ten different organs or tissues. The heat map was drawn in Log10-transformed expression values. The red or green colors represent the higher or lower expression level of each transcript in each sample. [file 12870_2020_2252_MOESM4_ESM.tif]

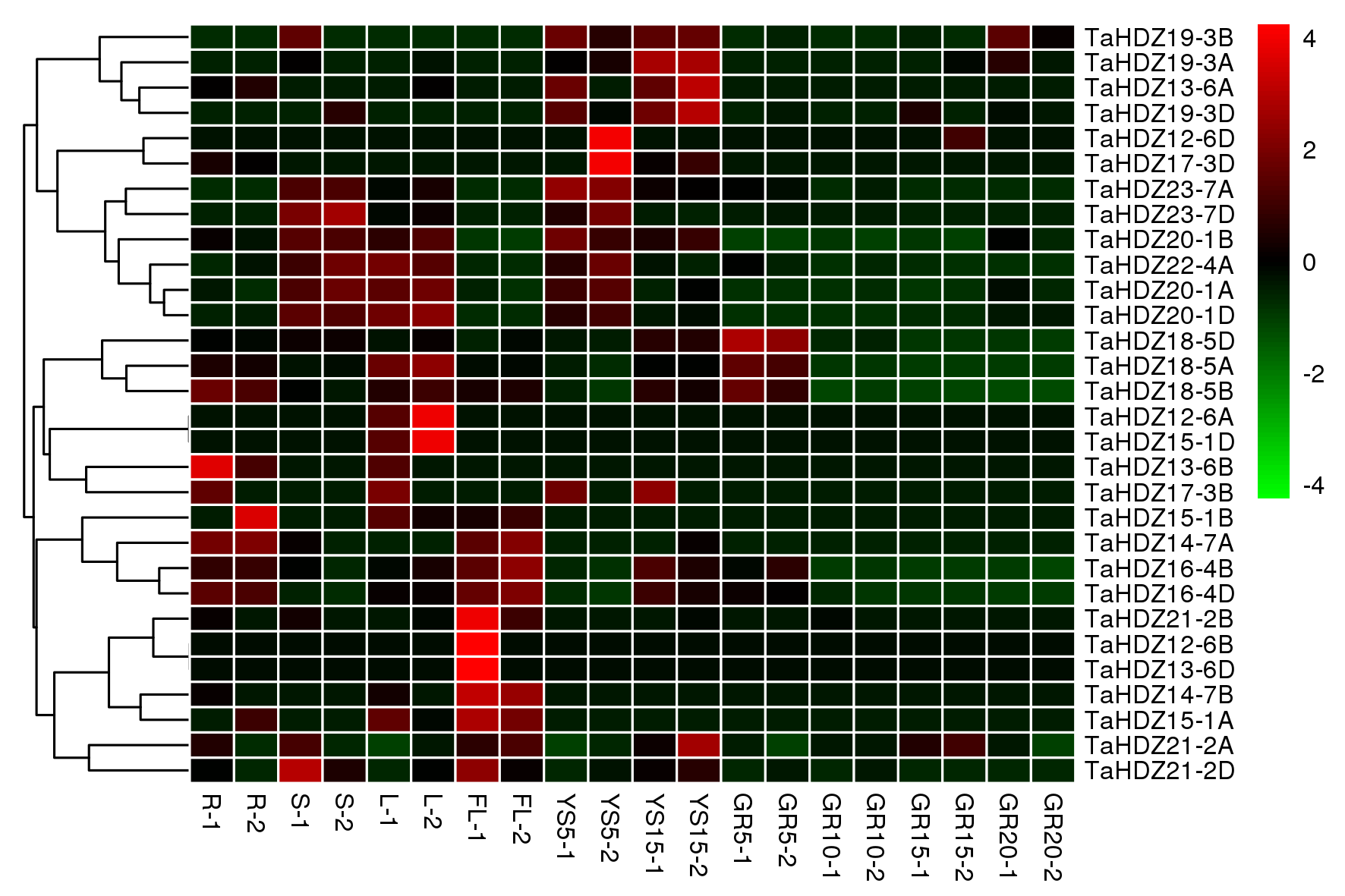

Supplement: Supplementary file 5 — Additional file 5: Figure S4. Hierarchical clustering of the relative expression level of family II TaHDZ genes in ten different organs or tissues. The heat map was drawn in Log10-transformed expression values. The red or green colors represent the higher or lower expression level of each transcript in each sample. [file 12870_2020_2252_MOESM5_ESM.tif]

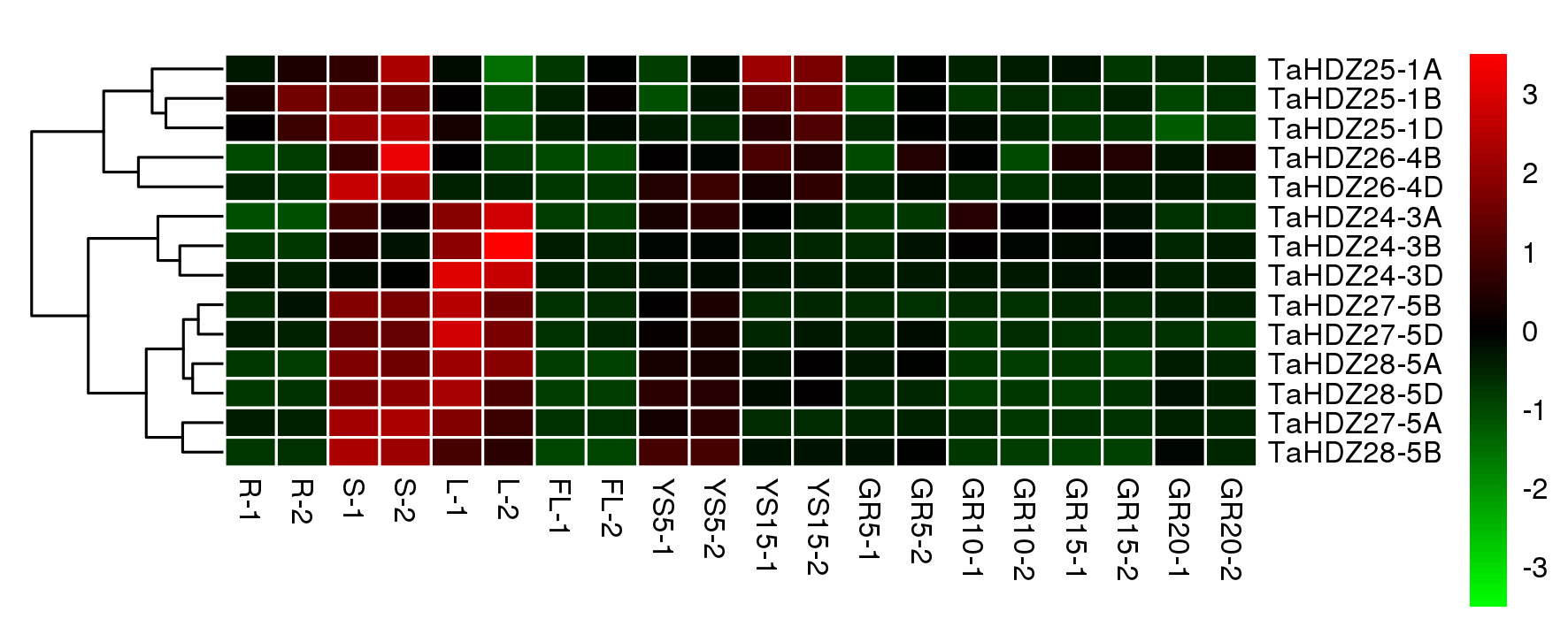

Supplement: Supplementary file 6 — Additional file 6: Figure S5. Hierarchical clustering of the relative expression level of family III TaHDZ genes in ten different organs or tissues. The heat map was drawn in Log10-transformed expression values. The red or green colors represent the higher or lower expression level of each transcript in each sample. [file 12870_2020_2252_MOESM6_ESM.tif]

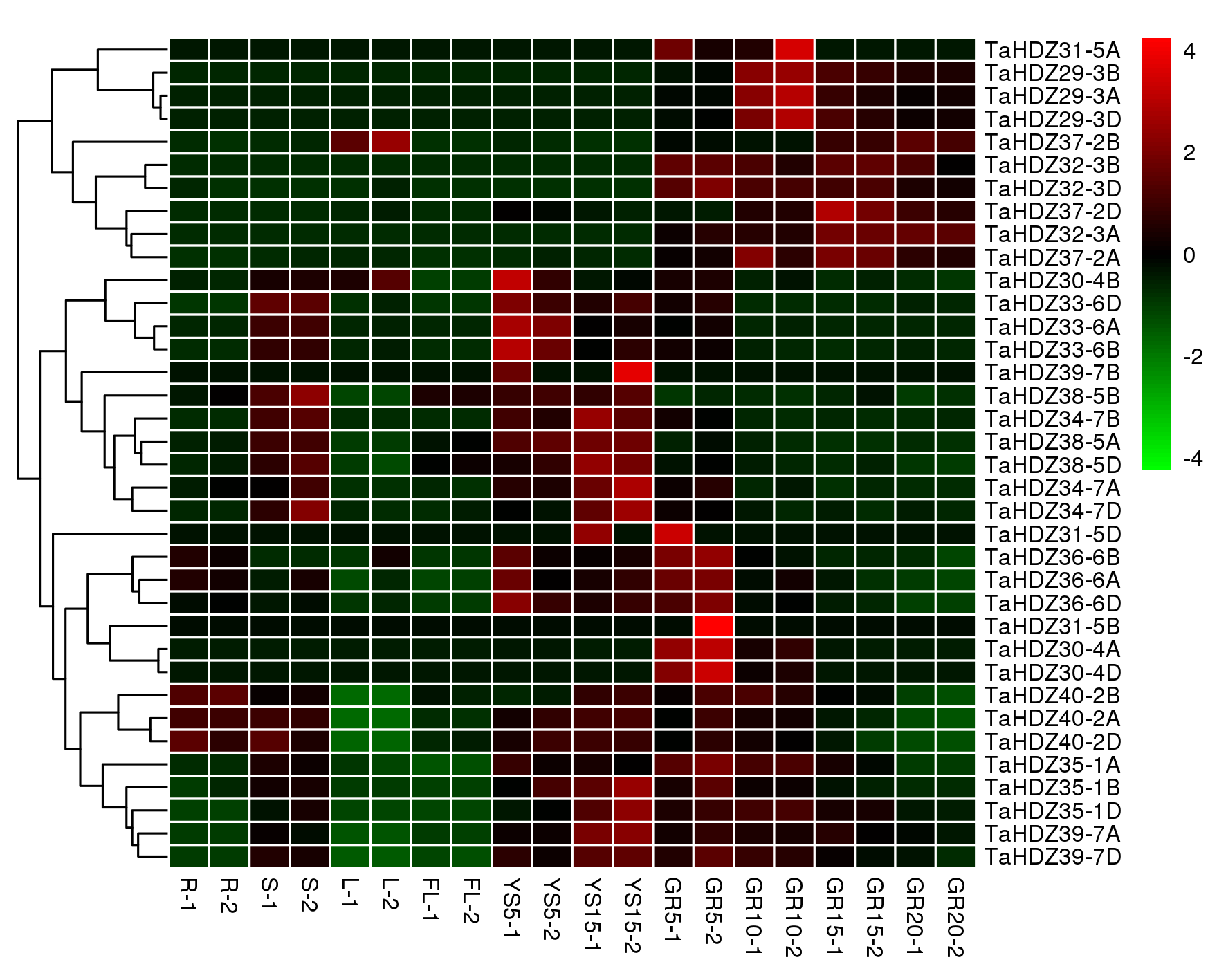

Supplement: Supplementary file 7 — Additional file 7: Figure S6. Hierarchical clustering of the relative expression level of family IV TaHDZ genes in ten different organs or tissues. The heat map was drawn in Log10-transformed expression values. The red or green colors represent the higher or lower expression level of each transcript in each sample. [file 12870_2020_2252_MOESM7_ESM.tif]

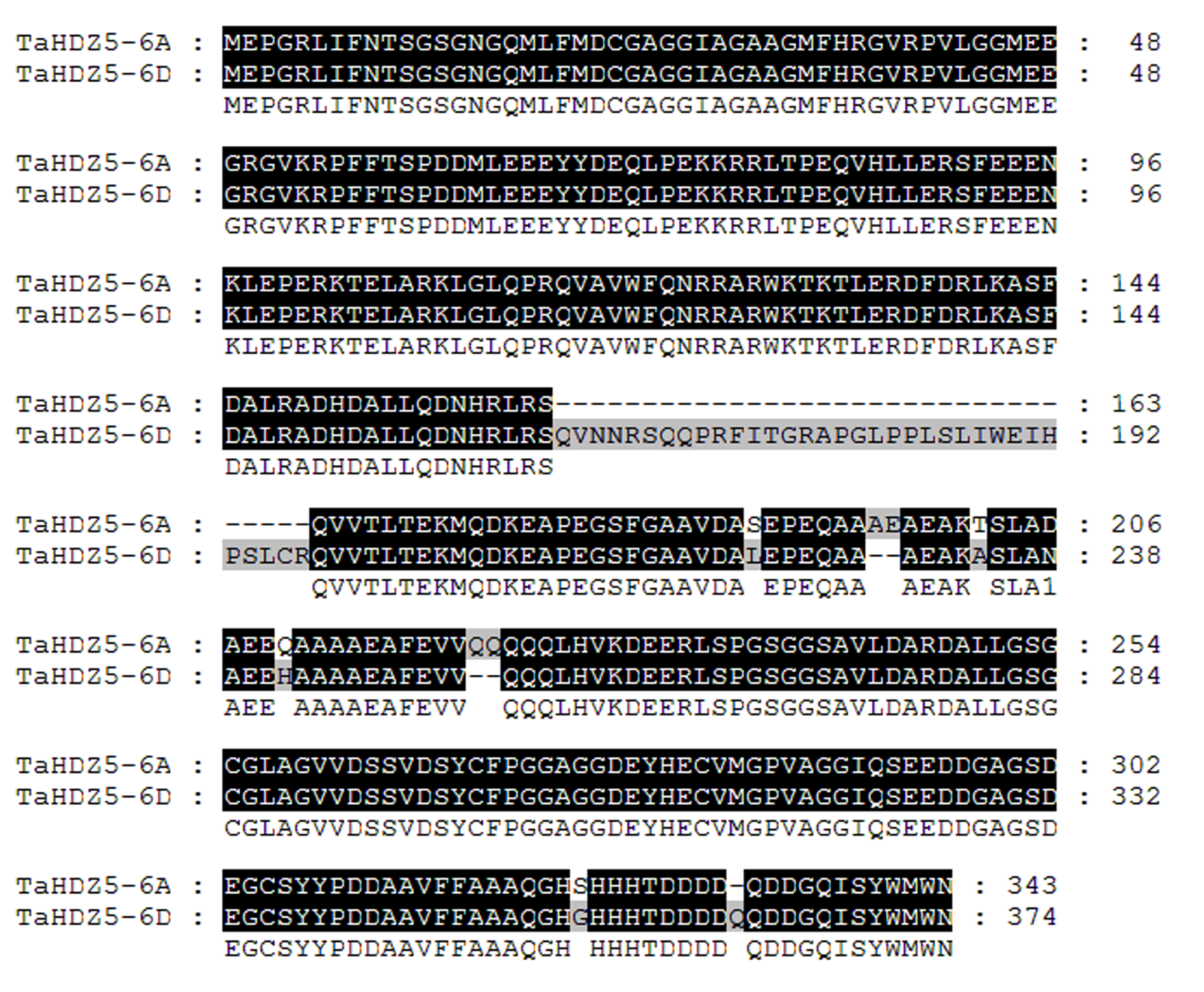

Supplement: Supplementary file 9 — Additional file 9: Figure S7. Protein sequence alignment of TaHDZ5-6A and TaHDZ5-6D. Identical amino acids are shaded in black, and similar amino acids are shaded in gray. [file 12870_2020_2252_MOESM9_ESM.tif]

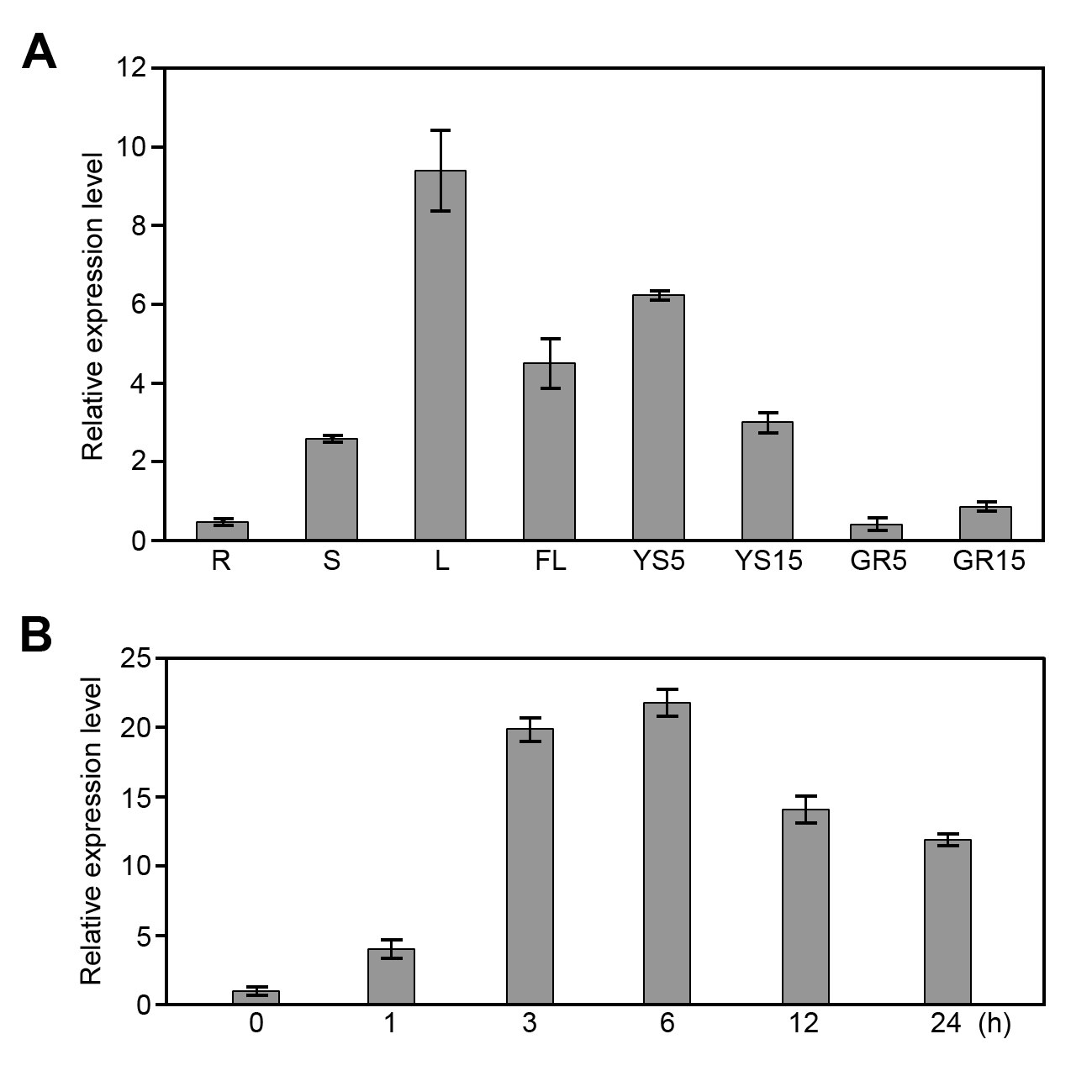

Supplement: Supplementary file 10 — Additional file 10: Figure S8. The expression patterns of TaHDZ5 in wheat. A The expression profiles of TaHDZ5 in different tissues. R, root of wheat seedling at five-leaf stage; S, stem of wheat seedling at five-leaf stage; L, leaf of wheat seedling at five-leaf stage; FL, flag leaf at heading stage; YS5, young spike at early booting stage; YS15, spike at heading stage; GR5, grain of 5 days post-anthesis; GR15, grain of 15 days post-anthesis. B The expression pattern of TaHDZ5 under drought stress treatment. The error bars indicate standard deviations derived from three independent biological experiments. [file 12870_2020_2252_MOESM10_ESM.tif]
